# Noninvasive ultrasound stimulation of the spleen to treat inflammatory arthritis

**DOI:** 10.1038/s41467-019-08721-0

**Published:** 2019-03-12

**Authors:** Daniel P. Zachs, Sarah J. Offutt, Rachel S. Graham, Yohan Kim, Jerel Mueller, Jennifer L. Auger, Nathaniel J. Schuldt, Claire R. W. Kaiser, Abigail P. Heiller, Raini Dutta, Hongsun Guo, Jamu K. Alford, Bryce A. Binstadt, Hubert H. Lim

**Affiliations:** 1 Department of Biomedical Engineering, University of Minnesota, Minneapolis, 55455 MN USA; 2 Restorative Therapies Group, Medtronic plc, Minneapolis, 55432 MN USA; 3 Center for Immunology and Department of Pediatrics, University of Minnesota, Minneapolis, 55455 MN USA; 4 Department of Otolaryngology-Head and Neck Surgery, University of Minnesota, Minneapolis, 55455 MN USA; 5 Institute for Translational Neuroscience, University of Minnesota, Minneapolis, 55455 MN USA

## Abstract

Targeted noninvasive control of the nervous system and end-organs may enable safer and more effective treatment of multiple diseases compared to invasive devices or systemic medications. One target is the cholinergic anti-inflammatory pathway that consists of the vagus nerve to spleen circuit, which has been stimulated with implantable devices to improve autoimmune conditions such as rheumatoid arthritis. Here we report that daily noninvasive ultrasound (US) stimulation targeting the spleen significantly reduces disease severity in a mouse model of inflammatory arthritis. Improvements are observed only with specific parameters, in which US can provide both protective and therapeutic effects. Single cell RNA sequencing of splenocytes and experiments in genetically-immunodeficient mice reveal the importance of both T and B cell populations in the anti-inflammatory pathway. These findings demonstrate the potential for US stimulation of the spleen to treat inflammatory diseases.

## Abstract (web summary)

Modulation of the cholinergic pathway and spleen function can reduce inflammation with invasive implants. Here, the authors show that non-invasive ultrasound stimulation of the spleen reduces disease severity in a mouse model of inflammatory arthritis, partly via altering B and T cell function.

## Introduction

**Fig. 1:**
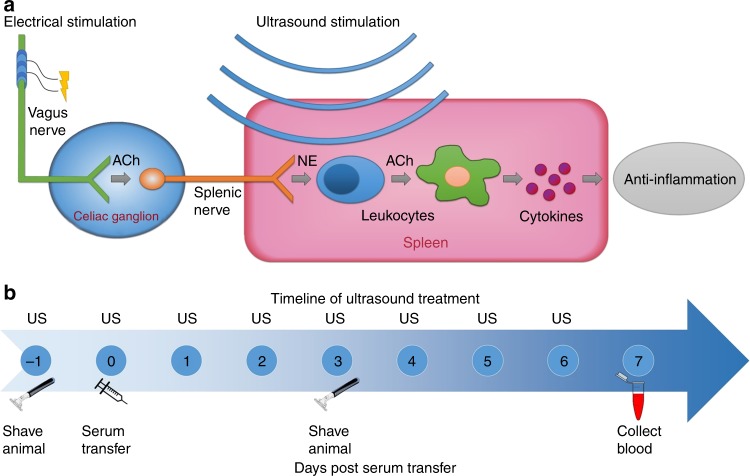
Modulation of the cholinergic anti-inflammatory pathway through the vagus nerve, splenic nerve and spleen. **a** Electrical stimulation of the vagus nerve or US stimulation of the spleen is thought to modulate the neural communication with T Cells and Macrophages, blocking the production of inflammatory cytokines and promoting an anti-inflammatory state. **b** Timeline of a typical experiment performed in the presented study in which animals were injected with 300 μl of K/BxN serum on day 0 and treated with focused US that targeted the spleen on days -1 through 6

Since its discovery in 2000, the cholinergic anti-inflammatory pathway has been extensively studied because of its role in modulating the mammalian immune response^1–3^. This pathway relies on a robust neural-immune interaction in which peripheral nerves communicate with and can alter the activity of the immune system. The proposed mechanism postulates that in response to infection or injury, the parasympathetic vagus nerve transmits signals from the brain to the adrenergic splenic nerve, which interacts with splenic immune cells (Fig. 1a). When the vagus nerve is experimentally stimulated with electrical current, this neural-immune reflex is triggered, dampening the inflammatory response to infection or tissue injury^4^. This pathway requires the interaction of the vagus nerve, splenic nerve, spleen, and splenocytes^5–7^. Vagus nerve stimulation (VNS) has been shown to reduce in vivo cytokine production during endotoxemia in rat and mouse models, including a significant reduction of tumor necrosis factor (TNF), interleukin-1 (IL-1) and other inflammatory cytokines. VNS has also been used to treat arthritis in animal models^8^, and there is a reported direct link between the cholinergic nervous system and the inflammatory process in inflamed joints^9^. More recently, VNS was used to treat rheumatoid arthritis in human patients using implantable vagus nerve electrode cuffs^10^. These techniques have uncovered a powerful non-pharmacologic therapeutic option for chronic inflammatory diseases via electrical stimulation.

Recent reports demonstrated that noninvasive US energy delivered to the abdomen of mice diminished inflammation and tissue damage during renal ischemic reperfusion injury (IRI)^11^. These anti-inflammatory effects were mediated by the spleen, and animals lacking T and B lymphocytes did not achieve the same protection from renal damage. It was suggested that US stimulation may activate the same cholinergic anti-inflammatory pathway triggered by VNS (Fig. 1a). Splenocyte transfer studies indicated that the leukocytes harvested from US-treated spleens could confer protection from IRI when injected into naïve recipient mice^12^. This finding suggested that US transforms these splenocytes to an anti-inflammatory state and that the US stimulation approach may be used to prevent or treat inflammatory conditions in addition to experimentally induced renal IRI.

In the rapidly emerging field of autonomic neuromodulation, and in bioelectronics medicine in general, there is both growing excitement and increased skepticism about the possibility of using such techniques to enhance organ function or to treat disease. A critical step to move this new field forward scientifically and clinically is to demonstrate that therapy and the proposed mechanism(s) of action are dependent on specific stimulation parameters and targeted activation of specific structures or cells, while being consistent and repeatable across animal and human studies. With this in mind, we investigated whether US targeting of the spleen in mice could noninvasively modulate the immune system to treat inflammatory arthritis and directly measured the therapeutic effects across a range of stimulation parameters. We demonstrate that significant therapeutic effects are possible only with specific US parameters and that both US dose and duration impact treatment efficacy. Furthermore, targeting of the spleen is crucial in achieving these therapeutic effects, since US stimulation of other body locations is ineffective. In addition, US stimulation is less effective in mice lacking T and or B cells. Single cell RNA sequencing reveals that most genes differentially expressed in splenic lymphocytes in response to US stimulation are induced in arthritic but not in non-arthritic mice, suggesting a unique therapeutic US effect in the setting of inflammation. In further support of our findings in an inflammatory arthritis model, the companion paper by Cotero et al^13^. uses a rat LPS acute inflammation model and independently demonstrates similar effective US parameters. These stimulation parameters are also shown to drive neurotransmitter and cytokine changes within the spleen consistent with modulation of the cholinergic anti-inflammatory pathway, and this effect is not achievable with stimulation of other targets outside of the spleen. Overall, these complementary findings across independent studies and model systems reveal the specific and robust nature of US-induced anti-inflammatory effects and its potential as a new type of non-pharmacologic therapy.

## Results

### Splenic ultrasound stimulation reduces arthritis severity

We used the K/BxN serum-transferred model of inflammatory arthritis. This model allows consistent induction of distal, symmetric polyarthritis when serum from T cell receptor transgenic, spontaneously arthritic K/BxN animals is injected intraperitoneally into recipient mice. Like rheumatoid arthritis in humans, the K/BxN serum-transferred arthritis causes inflammation due to IgG autoantibodies present in the serum^14^. Evaluation of the model in mice not exposed to US revealed considerable swelling in the ankles and paws in the days following serum transfer before naturally declining as the transferred antibodies were catabolized (Supplementary Fig. 1). Ankle thickness changes were measured with a caliper, and composite clinical scores were determined based on the established method of assessing rodent arthritis severity on a 0–12 scale; in brief, each paw was assigned a clinical score between 0 and 3, with 0 indicating no swelling as described in Methods^15,16^. Change in bodyweight was also monitored over the course of the study.

To investigate the effect of US treatment, mice were briefly anesthetized and treated noninvasively with spleen-targeted US or sham-US (control; same setup as treatment group except no energy delivered through the US transducer). We characterized the US pressure beam profiles, murine spleen dimensions, and spleen location to target the spleen using specific anatomical landmarks (Supplementary Fig. 2, 3 and Supplementary Table 1). Beginning one day prior to serum transfer, we administered daily US for 7 consecutive days (Fig. 1b), since the clinical score and ankle thickness tended to peak between day 7 and 10 (Supplementary Fig. 1). Each animal was monitored daily to document arthritis severity, and blood was collected on the final day of the experiment to validate successful transfer of arthritogenic antibodies (see Fig. 1b and Supplementary Methods).

**Fig. 2:**
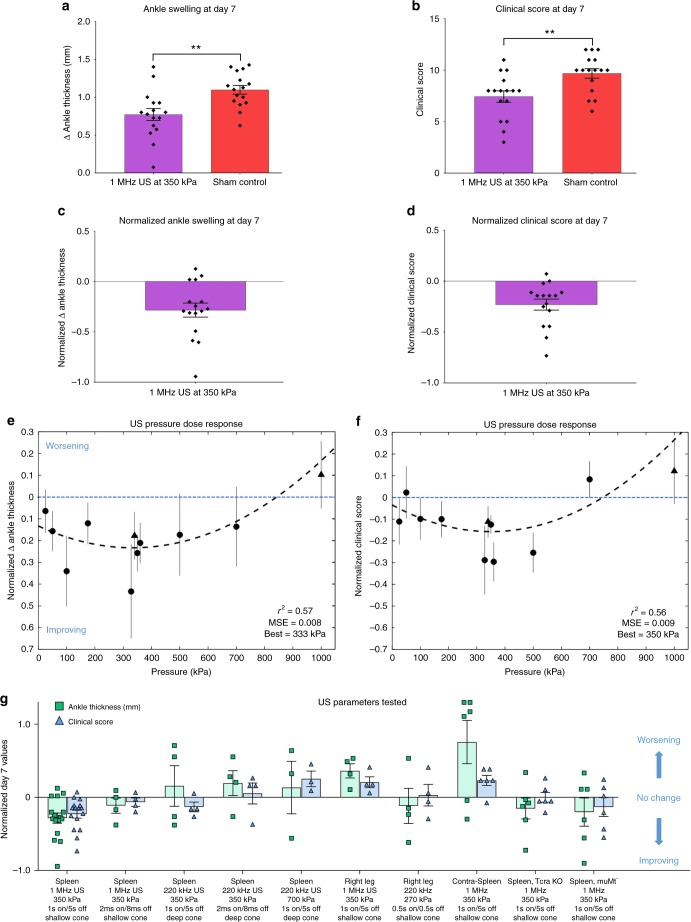
US treatment modulates arthritis severity. Results from 7-day arthritis experiments are presented. **a**–**d** Pooled data from three experiments (total *n* = 32 mice), using identical experimental conditions (1 MHz US at 350 kPa, 1 s on/5 s off bursts for 2 min per day; shallow US-focusing cone to target the spleen, see Supplementary Fig. 2a) are shown. On the final day of the experiment, change in ankle thickness (**a**) and clinical score (**b**) of US treated animals are significantly reduced compared to sham-US controls (*p* = 0.0017 and *p* = 0.0060, respectively, using a Mann–Whitney Test). For comparison, normalized data from the same set of experiments are shown in **c**, **d**. A normalized value of zero indicates that the animal’s arthritis outcome was similar to the average untreated (sham-US) animal, a positive value indicates clinical worsening, and a negative value indicates clinical improvement. **e**, **f** Dose-response curves of US pressure reveal an optimal US amplitude between 333–350 kPa. Each point represents the normalized mean change of US treatment compared to sham at day 7. 1 MHz US stimulation with 1 s on/5 s off bursts for 2 min per day was applied in each experiment. Circles indicate that the shallow US-focusing cone was used, and triangles indicate that the deep US-focusing cone was used. Results are pooled from 108 mice across 9 experiments (see Mice section of Methods). **g** Additional US stimulation parameters and body locations were also tested, revealing consistent and effective therapeutic effects when specifically using 350 kPa, 1 s on/5 s off bursts with the shallow US-focusing cone targeting the spleen. One hundred and three mice were used over 9 experiments. All conditions consisted of 2 min of US stimulation per day, except for the right leg conditions that consisted of 4 min per day. Contra-spleen refers to right abdominal stimulation contralateral to the spleen. Tcra KO and muMt^-^ were spleen stimulation experiments performed in T cell and B cell knockout mice, respectively. Mean and SEM are shown for all figures. Double asterisks denote *p* < 0.01. kPa kilopascal

**Fig. 3:**
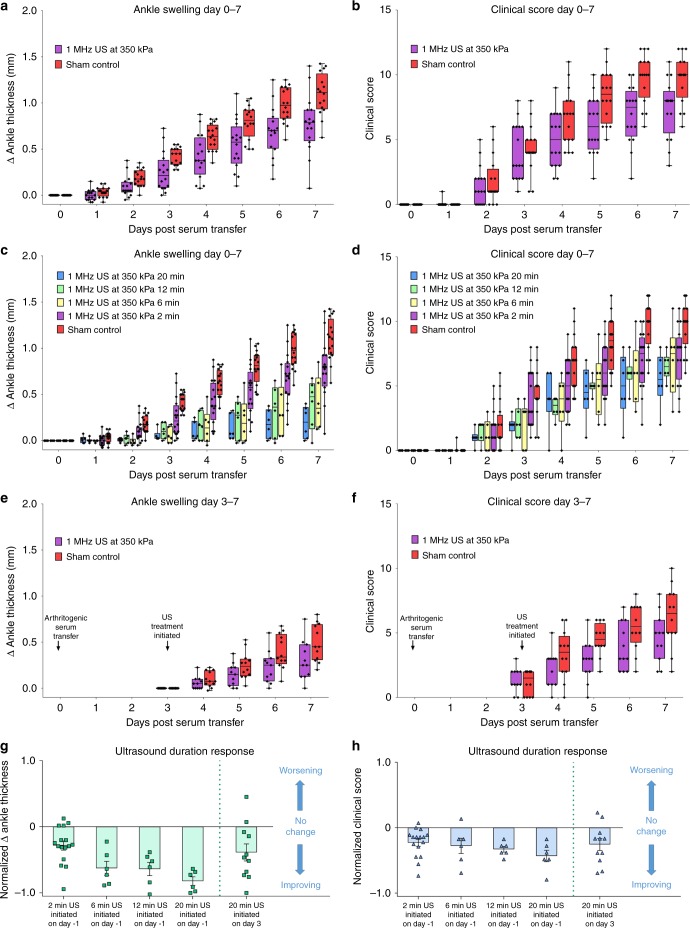
US treatment is effective before or after arthritis onset and depends on duration of stimulation period (page 18). All experiments in this figure used the 1 MHz US at 350 kPa, 1 s on/5 s off bursts in animals with arthritic serum transfer at day 0. **a**, **b** Full 7-day time course pooling data from the three experiments shown previously (Fig. 2a, b), which used identical experimental conditions for 2 min per day with US administered daily from day -1 through 6 (total *n* = 32). **c**, **d** Ultrasound duration causes a dose-dependent effect on arthritis. 6, 12, and 20 minute US stimulation was tested and is shown with the 2 min US and sham results from **a**, **b** for comparison. Figures show the 7-day progression of the disease and mice were stimulated with US from day -1 through day 6. For the 6, 12, and 20 min experiments, there were 6 animals per cohort (total *n* = 18) and for the 2 minute US and control data there were 16 animals per cohort (total *n* = 32). **e**, **f** US treatment is also effective at reducing symptoms after onset of arthritis. Treatment was not initiated until day 3 after arthritis had manifested. US was administered for 20 min per day. Data was pooled from two experiments with a total *n* = 23. **g**, **h** Normalized 7th day values of ankle thickness (**g**) and clinical score (**h**) for the different durations of ultrasound treatment shown in 3a–f. Error bars are SEM. 6, 12, and 20 minute values were normalized to the pooled sham control data from **a**, **b**. The bar “20 min US initiated on day 3” shows the improvement in ankle thickness and clinical score compared to the first day of treatment on day 3. In **a**–**f**, whisker plots are presented for better visualization of the data over time in which the endpoints of the vertical lines span the minimum to maximum values, the midline of each box is the median value and each box extends from the 25th to 75th percentile. In **g**, **h**, mean and SEM are plotted for each condition. kPa kilopascal

Daily splenic US treatment with a 1 MHz transducer presented at 350 kPa for 2 min per day reduced the ankle thickness and clinical score by the conclusion of the 7-day experiment (Fig. 2a–d). We used 1 s on/5 s off US pulse pattern for our initial studies, because this was shown to have anti-inflammatory effects by Gigliotti et al.^11,12^. Several different pressures were evaluated, but 350 kPa demonstrated consistent therapeutic effects (see next section). Specifically, compared to sham-treated animals, US treatment significantly improved final day ankle swelling (*p* = 0.0017) and clinical scores (*p* = 0.0060) using a Mann–Whitney test. The progression of arthritis development and the effects of US treatment over a 7-day period can be observed in Fig. 3a, b, in which US stimulation gradually improved ankle swelling and clinical scores over time, which was consistently observed across multiple experiments (Supplementary Fig. 4). In order to objectively compare results across separate experiments that employed different US stimulation parameters and arthritogenic serum batches, the ankle swelling and clinical scores were normalized (Fig. 2c, d; see Methods). A normalized representation of the data from Fig. 2a, b is shown in Fig. 2c, d with positive values indicating a worsening of arthritis and negative values representing an improvement of arthritis. We further used this normalization method to optimize US treatment by comparing the therapeutic effects across different US parameters (carrier frequency, pressure amplitude, focal depth, and duration) and conditions (knock-out mice lacking T or B cells and treatment initiated before or after onset of arthritis) described below.

### Identifying the optimum ultrasound pressure dose

We assessed whether the effect of US on arthritis severity depended on the pressure amplitude of stimulation by evaluating US pressures ranging from 25–1000 kPa, all delivered by a 1 MHz US transducer at 1 s on/5 s off bursts for 2 minutes per day. The normalized dose-response curves for US pressure amplitude and effect on arthritis severity are shown in Fig. 2e, f. These results reveal a U-shaped pressure dose response curve with an optimal US pressure of between 333–350 kPa, and suggest that arthritis-ameliorating effects depend on specific US pressure amplitudes.

### Identifying therapeutic parameters and targeting

We investigated additional US parameters including acoustic carrier frequency, pulse pattern and focal depth, as well as target location. Stimulation of the spleen at an acoustic frequency of 220 kHz was not as effective at reducing arthritis as stimulation at 1 MHz, and the 1 s on/5 s off pulse pattern (16.7% duty cycle) was consistently more effective than the 2 ms on/8 ms off (20% duty cycle) pulse pattern when using 1 MHz (Fig. 2g). Custom-built US-focusing cones with different beam focal depths were compared (Supplementary Fig. 2). The shallow focusing cone was more effective at reducing the symptoms of arthritis (Fig. 2g), and was therefore used for a majority of the studies. We identified an effective treatment parameter setting using the 1 MHz frequency at 350 kPa with a 1 s on/5 s off pulse pattern while targeting the spleen with the shallow-focusing cone. We performed three separate experiments using this parameter and observed consistent therapeutic effects (Fig. 3a, b and Supplementary Fig. 4). All data points from those three experiments are pooled together and plotted in Fig. 2g in the left−most position (same data as in Fig. 2c, d). Stimulating other body areas such as the anterior and posterior thigh regions (Fig. 2g, Right Leg) or on the contralateral abdomen opposite to the spleen (Fig. 2g, Contra-Spleen) did not effectively reduce arthritis severity.

Daily US stimulation duration had a dose-dependent effect on arthritis (Fig. 3c, d). We tested 6-, 12-, and 20-minute US durations using the same parameters as before with a 1 MHz stimulus calibrated to 350 kPa and using a 16.7% duty cycle. Animals from the 6-, 12-, and 20-minute cohorts were handled identically and underwent the same dose of anesthesia per day (20 min of 1.5% isoflurane). Longer durations of treatment led to greater reductions in arthritis severity. Normalized data presented in Fig. 3g, h display this inverse relationship; notably, half of the animals receiving 20 min of daily US treatment exhibited almost no ankle swelling (values approaching −1). This duration dose-dependent trend is statistically significant for ankle thickness and clinical score, as shown in the Supplementary Fig. 5, which plots the linear regression of the response curve (*p* < 0.0001, *R*^2^ = 0.3839 and *p* = 0.0460, *R*^2^ = 0.1187 for ankle thickness and clinical score, respectively). Longer duration of US stimulation was also significantly correlated with reduced disease-driven weight loss (Supplementary Fig. 6).

### Therapeutic effect of ultrasound depends on lymphocytes

As described in the Introduction, previous studies have proposed that splenic T and B cells contribute to the anti-inflammatory effects induced by electrical stimulation of the vagus nerve or by US stimulation of the spleen^1–12^. To investigate if T and B cells contribute to the arthritis-ameliorating effects of splenic US stimulation in our experiments, we performed two additional experiments in mice genetically lacking T or B cells. Splenic US did not consistently reduce arthritis severity in T cell or B cell deficient mice (Fig. 2g, “Tcra KO” and “muMt−”, respectively), suggesting that the anti-inflammatory effect of US in our model similarly depends on lymphocytes, either directly or indirectly. Interestingly, a few animals in both experiments did exhibit some improvement in inflammation, suggesting that the therapeutic mechanism may involve additional cell types.

### Ultrasound can be used to prevent or treat arthritis

The results presented thus far involve initiation of ultrasound prior to induction of arthritis. We next tested whether US delivered after the onset of arthritis could still produce therapeutic effects. This is directly relevant for clinical applications since patients seek medical treatment after the onset of arthritis. All animals were injected with arthritogenic serum on day 0 but were otherwise undisturbed to allow arthritis to progress until day 3. On the third day, US or sham-US was initiated. We observed improvements in disease severity up to day 7, in which ankle thickness in the US cohort was significantly improved (*p* = 0.0490) and clinical score also trended toward improvement (*p* = 0.0900) using a Mann–Whitney test, as shown in Fig. 3e, f. The treatment was consistent across two independent experiments (total *n* = 23; Supplementary Fig. 7). For comparison with the results for the other duration parameters, these data are normalized to the average sham data from the same experiment and plotted in Fig. 3g, h. These experiments demonstrate that US stimulation targeting the spleen after onset of arthritis is also capable of improving the progression of the disease.

### Single-cell RNA sequencing in ultrasound treated animals

We have demonstrated that the efficacy of US depends on specific targeting of the spleen, and that efficacy is reduced in animals lacking T or B cells. We therefore sought to determine the molecular mechanisms underlying the reduced joint inflammation seen with US treatment by investigating the gene expression profiles of lymphocytes in the spleen after 7 days of US therapy using single cell RNA sequencing (scRNA-seq). Spleen-targeted US or sham-US was delivered by a 1 MHz US transducer at 1 s on/5 s off bursts for 12 min per day, which was a clinically effective stimulation duration in our previous experiments and still enabled us to evaluate 16 mice within a reasonable time period per day. We compared four treatment groups: arthritogenic serum-injected mice that received sham-US or US treatment and control non-arthritogenic serum-injected mice that received sham-US or US treatment, with four animals in each group (*n* = 16, Supplementary Fig. 8b). For this analysis, CD45 + splenic leukocytes from the four mice in each treatment group were combined, and ~25,000 cells per treatment group were analyzed by scRNA-seq. Cells were filtered, normalized, and analyzed for heterogeneity in transcriptional profiles, and cell types were determined and assigned based on well-established cell-type markers. Here, we analyzed T cells or B cells for each treatment group. Cells were assigned by unbiased clustering based on a principal component analysis and expression of *Cd3g* (encoding CD3g) and *Ms4a1* (encoding CD20) transcripts, which are known markers for T and B cells, respectively.

**Fig. 4:**
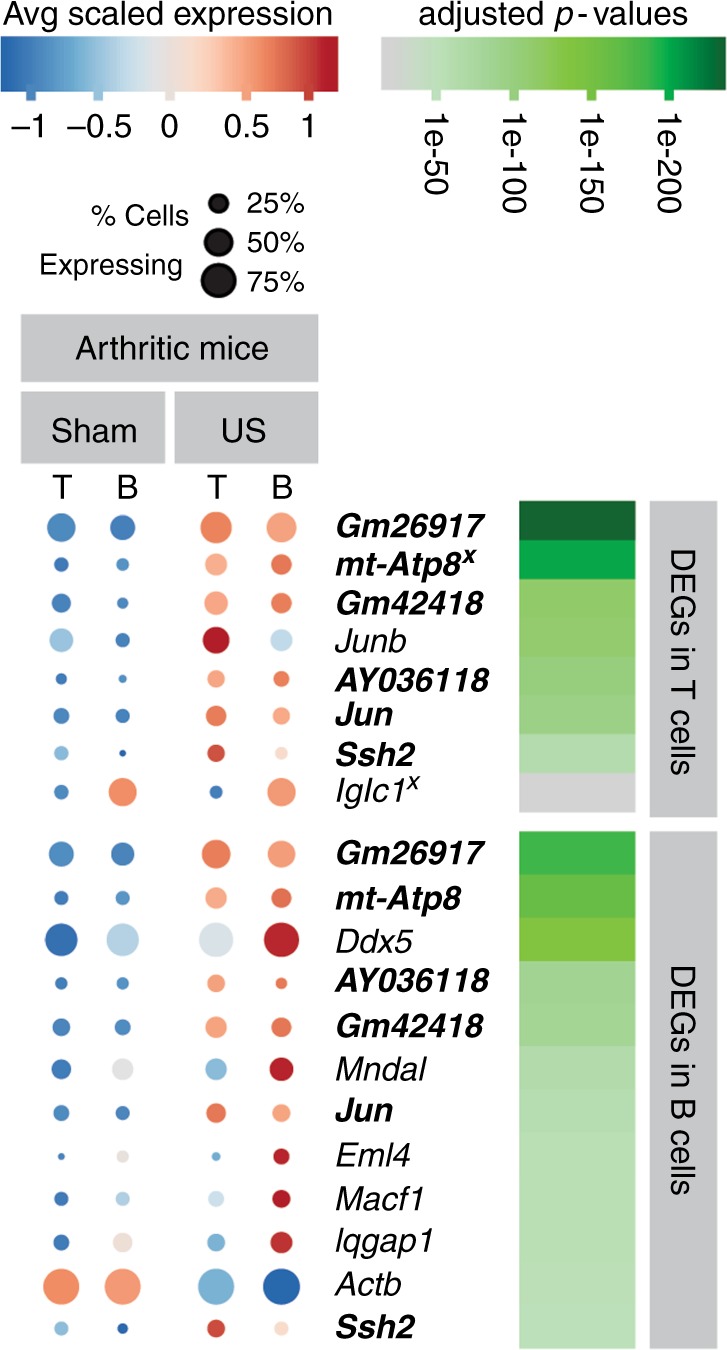
Splenic T and B cells demonstrate induction of genes following US treatment. The dot plot displays the statistically significantly differentially expressed genes (DEGs) in T cells (top list) and B cells (bottom list) from mice given arthritogenic serum comparing US versus sham-US stimulation. The size of each circle represents the percent of cells within each cell type (T cells = T, B cells = B) which express the gene listed, and the color of each circle represents average scaled expression. Gray bars denote mouse treatment groups; genes that are in bold are statistically significantly DEG in both T cells and B cells, and genes found to also be DEG in non-arthritic mice with US stimulation (see Supplementary Fig. 8) are denoted with a superscript x. Adjusted *p*-values for each gene in either T or B cells (top or bottom gene lists) are shown in shades of green. All DEGs listed in this Figure range in statistical significance from *p* = 2.39e-02 to *p* = 3.95e-230 using a Wilcox rank sum test with Bonferroni correction

From this scRNA-seq data, we determined which genes were differentially expressed due to US stimulation. Among mice that received arthritogenic serum, we analyzed changes in gene expression in T and B cells that could be attributed to US treatment. The differentially expressed genes (DEGs) are shown in Fig. 4. We similarly identified DEGs in non-arthritogenic serum-injected mice (subjected to US or sham-US treatment; Supplementary Fig. 8a). In T cells from arthritic mice, we identified 8 DEGs with adjusted *p*-values <0.05 (ranging from *p* = 3.95e-230 to 5.04e-45 for the first seven genes, and *p* = 0.0239 for Iglc1 using a Wilcox rank sum test with Bonferroni correction) when comparing US-treated versus sham-US mice (Fig. 4 and Supplementary Fig. 8b). Similarly, we found 12 DEGs (US versus sham-US) in B cells in arthritic mice (adjusted *p*-values ranging from 9.76e-179 to 1.75e-34 using a Wilcox rank sum test with Bonferroni correction), and all but one were upregulated with US treatment (Fig. 4). Adjusted *p*-values show robust gene expression differences; however, only modest average log fold changes (logFCs) existed between the two treatment groups (Supplementary Table 2). These modest logFCs could result from a low percentage of cells expressing a particular gene or could indicate that the ultrasound treatment induces a relatively minor perturbation to the immune system. Interestingly, most of the DEGs found in arthritic mice were not differentially expressed in the non-arthritic mice (except mt-Atp8 and Iglc1 denoted with x in Fig. 4, Supplementary Fig. 8), suggesting a unique effect of US stimulation on arthritic mice. Additionally, 6 out of the 8 DEGs (bolded genes in Fig. 4) were upregulated in both T and B cells in arthritic mice, suggesting US induces overlapping effects in these two cell types. These findings demonstrate that US stimulation targeting the spleen induces significant changes in the transcriptional profiles of lymphocytes, and interestingly these changes are unique to the arthritic disease state. Our finding that genes in T and B cells are significantly differentially expressed with US treatment is consistent with literature showing these cell types to be involved in the splenic anti-inflammatory pathways^4,7**–**9^.

## Discussion

Overall, our investigation into the therapeutic potential of noninvasive US targeting of the spleen has revealed that US can be used to reduce the severity of serum-transferred inflammatory arthritis in mice. We report that this therapy is effective when initiated either before induction of arthritis or after the onset of disease. Furthermore, we identified optimal US treatment parameters (e.g., 1 MHz and 1 s on/5 s off pulse pattern) and intensities (e.g. ~350 kPa) and demonstrated that longer US stimulation durations achieve greater therapeutic effects. Similar findings have also been demonstrated in our companion paper by Cotero et al^13^., which investigated US stimulation of the spleen using a 1.1 MHz transducer. Their results indicated an effective intensity range between 250-830 kPa that led to greater modulation of the anti-inflammatory response in a rat model of lipopolysaccharide-induced inflammation compared to other parameters and stimulation locations. Similar to our results, they also observed a U-shaped pressure dose response curve.

The exact mechanism(s) underlying US modulation of the splenic anti-inflammatory pathway is unknown. Several studies have shown that US stimulation can activate or modulate peripheral nerves^17–19^, possibly through a mechanical or thermal effect that actuates or modulates voltage-gated ion channels or mechanosensitive ion channels on neural tissue membranes, or through a cavitational effect resulting in direct ionic flux^19–21^. US can also activate skin receptors in humans and other excitable cell types, or induce cell membrane porosity^22–24^. Therefore, US targeting of the spleen could activate or modulate the splenic nerve axons and/or terminals though a mechanical or cavitational effect. These US manipulations could also potentially activate receptors of other cells within the spleen that need to be further investigated. Although the US pressures (~350 kPa) and pulse repetition patterns (1 s on/5 s off) shown to be effective in our study would lead to a negligible rise in gross tissue temperature (see Supplementary Fig. 9), it remains possible that a sufficient localized increase in temperature could change the electrical capacitance of the cell membrane or activate heat-sensitive ion channels along the splenic nerve fibers or terminals, based on previous modeling and in vitro studies^25,26^.

To gain insight into the molecular mechanism underlying these effects, we used scRNA-seq, which revealed genes in T and B cells whose expression is upregulated following US treatment in arthritic mice. We identified genes encoding transcriptional regulators such as Jun (c-Jun) and Junb (JunB), and also long non-coding RNAs such as Gm26917, Gm42418, and AY036118, which are upregulated with US treatment. Many of these genes, along with Ssh2, which encodes a phosphatase involved in actin filament depolymerization, are also upregulated in both T and B cells. The proteins c-Jun and JunB (among other proteins including Fos) can dimerize to form the transcription factor AP-1^27^, which impinges on multiple inflammatory pathways^28,29^. Additionally, we identified other genes involved in microtubule formation and crosslinking (Ssh2, Eml4, and Macf1) that were significantly upregulated in B cells, whereas Actb encoding β-Actin was significantly down regulated. Thus, another possibility is that altered expression of genes involved in regulation of the cytoskeleton affect lymphocyte polarization or migration, leading to reduced infiltration of these cells to synovium and inflammation upon US treatment^30^. Interestingly, we did not identify differential expression of genes encoding heat-shock proteins, which might be expected if US were causing a substantial increase in temperature or cellular stress. This finding is consistent with the minimal heating effects shown in the US simulation results presented in Supplementary Fig. 9.

Our single cell transcriptional analysis serves as a catalyst for investigating anti-inflammatory mechanisms with US treatment. Previous studies support a model by which vagus nerve stimulation causes T cells to release acetylcholine, resulting in the suppression of inflammatory cytokines produced by macrophages bearing α7 nicotinic acetylcholine receptors^4^. Furthermore, increased vagus nerve signaling leads to the aggregation of B cells in the marginal zone (MZ) of the spleen. In fact, vagus nerve stimulation resulting in MZ-accumulation of B cells requires intact splenic innervation, suggesting that the anti-inflammatory pathway also controls splenic immune cell trafficking^31^, although it is not yet clear how altered B cell trafficking would impact the K/BxN serum-transferred arthritis model. T and B cells play a crucial role in this cholinergic anti-inflammatory pathway, and our transcriptome-wide data reveal that T and B cells are transcriptionally modulated upon US stimulation. It remains to be determined if the differentially regulated genes in these cell types have any downstream effect on the cholinergic anti-inflammatory pathway or potentially novel pathways that result in the anti-inflammatory effect.

Although the findings presented in this study were consistent across experiments, we observed some variability in therapeutic outcomes across animals. There is biologic variability of arthritis severity in both treated and untreated animals, as is typical of models of inflammatory arthritis and disease states in humans. Additional variability in treatment outcomes may be attributed to anatomical differences across animals that resulted in variations in targeting of the spleen with US energy, although we confirmed a consistent location of the spleen across multiple animals (see Supplementary Fig. 3). Future development of an US transducer that can both image and focus stimulation energy to the spleen in real-time could enable more accurate targeting, and thus greater and more consistent therapeutic outcomes.

One key clinical advantage of US for human implementation is that it can noninvasively target nerves and cells within the spleen. Implanting electrodes into the spleen can damage the surrounding tissue and attaching cuff electrodes around very small diameter splenic nerve fibers near the spleen is clinically and technically challenging. Although cuff or penetrating electrodes can be implanted around the larger vagus nerve bundle in the neck region, this procedure requires invasive surgery and can lead to activation of different fiber types that project to numerous brain and body structures involved with multiple physiological and life-sustaining functions, including respiration^32^, digestion^33^, and maintaining heart rate^34^. Studies in animals and humans have reported various side effects associated with direct vagus nerve stimulation, including pain, dyspnea, or temporary facial paresis^35^. More serious adverse effects such as irreversible nerve damage are rare but have been reported^36^. US stimulation provides a noninvasive approach towards activating or modulating nerves and cells within the spleen that could potentially minimize side effects and enhance therapy for various inflammatory diseases.

Due to the unique potential of US to provide a noninvasive, non-pharmacological approach to treating inflammatory diseases, as well as the consistency in results across studies between two independent research groups, we have initiated a pilot clinical trial (ClinicalTrials.gov Identifier: NCT03690466) funded by the United States Defense Advanced Research Projects Agency (DARPA) to explore the use of US as an alternative therapy for human patients with rheumatoid arthritis. Positive findings from this clinical trial, combined with the encouraging data presented in these companion papers, will open new opportunities for applying US of peripheral nerves and end-organs to treat a range of chronic inflammatory diseases, such as rheumatoid arthritis and other health conditions.

## Methods

### Mice

Mice were maintained under Institutional Animal Care and Use Committee-approved protocols at the University of Minnesota (protocols 1506-32700A, 1512-33239A and 1711-35284A) and we have complied with all relevant ethical regulations for animal testing and research. K/BxN transgenic mice were bred in specific-pathogen-free colonies and were used to produce arthritogenic donor serum. C57BL/6 J recipient mice were purchased from the Jackson Laboratory. Serum was transferred to recipient animals as described in the Supplementary Information. 276 mice were used in this report. For the US pressure dose response curves shown in Fig. 2e, f, a total of 108 animals were included in the normalization analysis (*n*_25_ = 5, *n*_50_ = 6, *n*_100_ = 6, *n*_175_ = 5, *n*_350_ = 21, *n*_500_ = 6, *n*_700_ = 6, *n*_1000_ = 4, *n*_Sham_ = 49).

### Arthritis assessment

The daily change in average ankle thickness was measured with a caliper (Kafer J 15 Thickness Gauge, 0.01 mm precision) as is standard in rodent models of arthritis^37^ and clinical scores were based on the established method of assessing rodent arthritis index on a 0–12 scale^15,16^. Each paw was assigned a clinical score between 0 and 3, with 0 indicating no swelling. Clinical score (per paw): 0 = no evidence of inflammation; 1 = minor inflammation (metatarsal phalanges joints, individual phalanx, or localized edema); 2 = moderate swelling but localized to either dorsal or ventral surface of paw; and 3 = highly inflamed swelling on all aspects of paw. Maximum score is 12. Change in bodyweight was also monitored daily over the course of the study using a digital scale (Ohaus Model CL 201, 0.1 g resolution). These assessments were not blinded.

### Normalization of ankle thickness, clinical score, and weight

To objectively analyze results across separately performed experiments that were executed using different US stimulation parameters and serum transfer batches, the ankle swelling, clinical scores, and change in bodyweight were normalized. Normalization was completed within each experiment by dividing each measured value by the mean of the sham group for that day. $N_{{\mathrm{Day7}}} = \frac{{V_{{\mathrm{Day7}}} - {\mathrm{Sham}}_{{\mathrm{day7mean}}}}}{{\left| {{\mathrm{Sham}}_{{\mathrm{day7mean}}}} \right|}}$, where *N* is the normalized value for a given animal, *V* is the measured value for a given animal, Sham_day7mean_ is the mean across animals for that experiment’s 7th day sham values.

### Statistical analysis

Statistical analysis for the arthritis treatment outcomes was performed using the program Prism 7 (GraphPad Software). An unpaired, nonparametric Mann–Whitney test was performed as indicated in the figure legends and text, and the data satisfied equal variance criteria. Means and standard error of the means (SEM) are indicated in the figures. Box-and-whisker plots were created using Prism’s min to max function in which the endpoints of the vertical lines span the minimum to maximum values, the midline of each box is the median value and each box extends from the 25th to 75th percentile. Sample size was determined based on initial experiments in which we saw significant differences between stimulation cohorts. *R*^2^ and *p* values were listed for linear regression as indicated in the figure legends and text and were calculated using a nonparametric Spearman correlation. Differential expression analysis of the RNA sequenced data was performed using the Wilcox rank sum test reported with an adjusted *p*-value applying the Bonferroni correction.

### Ultrasound stimulation

Single element US transducers were driven by a 100 W (E&I 2100 L) or 200 W (E&I 2200 L) amplifier. Carrier signals were delivered by a Keysight 33500B Series Waveform generator. The US transducers were focused using custom designed focusing cones filled with degassed water and coupled to the animal using clinical US gel.

### scRNA-seq sample preparation and cell sorting

scRNA-seq was performed on splenocytes from 16 mice from four different cohorts, receiving either arthritogenic or non-arthritogenic serum and with or without US treatment as described in the Supplementary Information. Mice were treated with either sham or US treatment for 7 days, and splenocyte single cell suspensions from each mouse spleen were prepared and stained. Splenocytes were then sorted for live, leukocyte (CD45+) cell populations that were immediately sent for scRNA-seq library preparation. See Supplementary Information for more details.

### Library preparation and scRNA-seq bioinformatics analysis

Sorted leukocytes from each animal were immediately processed using the 10X Genomics Chromium Single Cell 3’ Library & Gel Bead Kit (v2) and libraries were analyzed for cDNA content and size. Libraries were then run on an Illumina MiSeq Sequencer to determine number of UMIs (cells) per sample. We ran these samples on 2 lanes of the Illumina NovaSeq Sequencer, which were then mapped to the mm10 mouse genome using the 10X Genomics Cell Ranger pipeline. Further statistical analyses were performed using the Seurat software package for R^38^. See Supplementary Information for more details.

## Supplementary information

Supplementary Information

## Data Availability

The data that support the findings of this study are available from the corresponding authors upon reasonable request. Single-cell RNA sequencing data are accessible through Gene Expression Omnibus (GEO) repository under series accession number GSE125316.
