# Supplementary material for: Noninvasive ultrasound stimulation of the spleen to treat inflammatory arthritis
Source: Nat Commun. 2019 Mar 12;10:951. doi: 10.1038/s41467-019-08721-0 (PMC6414603; doi:10.1038/s41467-019-08721-0)
Supplement: Supplementary file 1 — Supplementary Information [file 41467_2019_8721_MOESM1_ESM.docx]

**Noninvasive ultrasound stimulation of the spleen to treat inflammatory arthritis**

*Supplementary Information*

Zachs *et al.*

**Supplementary Methods**

*Time Course of Arthritis without Treatment.* The time course of the progression of ankle swelling and clinical score is shown for animals without treatment in Supplementary Fig. 1. In Supplementary Fig. 1a,b, two separate arthritic cohorts of C57BL/6J recipient mice (male, age 6-8 weeks) were injected on day 0 and again on day 2 with 150 μl of K/BxN serum and each cohort received different batches of arthritogenic serum. The third cohort did not receive arthritic serum. This preliminary study was designed to test the reliability of different batches of serum. The protocol of two injections of 150 µl of serum on day 0 and 2 was based on the protocol used in prior studies (S1). In Supplementary Fig. 1c,d, C57BL/6J recipient mice (male, age 6-8 weeks) were injected with 300 μl of arthritogenic serum on day 0 and given daily sham-ultrasound (sham-US) treatment for 2 minutes per day. We attempted to minimize the confounding effects of stress and handling of the mice by using a single injection of 300 μl instead of two injections. We found that the single injection produces a similar disease response, and used this protocol for all experiments included in this report.

*Focused Ultrasound Characterization.* We tested a 1 MHz concave transducer (Olympus I8-0118-P-SU) with two depths, and a 220 kHz flat transducer (Sonic Concepts 071-02-SN01) with two depths using custom built focusing cones, shown in Supplementary Fig 2. Early testing indicated that the 1 MHz transducer paired with the shallow beam profile was consistently effective at treating arthritis and was subsequently used for the majority of tests in our investigation. Other parameters tested are shown in Fig. 2e-g. The 1 MHz transducer coupled with the shallow cone outputs a beam profile with full width at half maximum (FWHM) of 2.2 mm diameter at 1 mm from the cone tip. Hydrophone measurements in degassed water indicate that the normalized pressure amplitude of this setup measured at 5 mm from the cone tip is approximately 50% of the maximum pressure achieved and continues to decrease with further depth. Post-mortem dissection of mice used in the experiments identified that the murine spleen is situated just below the abdominal wall, less than 1 mm from the tip of the focusing cone (mean spleen depth = 0.95 ± 0.01 mm, Supplementary Table 1 below). This would suggest, based on the depth (Z dimension) of the US beam pressure profile, that more than 80% of the maximum US pressure amplitude is capable of reaching the spleen.

*Spleen Depth and Dimensions.* The spleen depth and dimensions were analyzed from eight mice and shown in Table 1. After CO_2_ euthanasia of eight animals used in the study, the skin and abdominal tissue directly over the spleen was retracted and the spleen was extracted. The surface of the spleen is in direct contact with the abdominal wall, so the depth (distance from the skin to the surface of spleen) was measured by placing the skin and abdominal tissue above the spleen in a precise calipers (Kafer J 15 Thickness Gauge, 0.01 mm resolution). The dimensions of the spleen itself were measured after extraction with a surgical ruler. The depth and dimensions of the mouse spleen are shown below.

*Experimental Setup and Spleen Targeting.* To allow consistent US treatment, ankle measurements and clinical scoring, the mice were anesthetized with 1.5% isoflurane during each session and placed on a temperature-controlled heating pad. All animals within an experiment received the same amount of time under anesthesia, regardless of whether they were in the US or sham cohort. Clinical scores and ankle thickness were also measured while each animal was anesthetized on days -1 to 6 (relative to day of serum injection), and final ankle thicknesses and clinical scores were measured just after CO_2_ euthanasia on day 7. Mice were shaved over their left flank on the first day of the experiment and the US focusing cone filled with degassed water was coupled to the skin with US gel. Sham controls followed the same procedure, but US was not turned on. Hair was shaved again on day 3 post-injection to allow maximum US penetration of the skin. The daily change in average ankle thickness was measured with a precise caliper (0.01 mm resolution) and clinical scores were based on the established method of assessing rodent arthritis index on a 0-12 scale (13, 14). Each paw was assigned a clinical score between 0 and 3, with 0 indicating no swelling. Clinical score (per paw): 0 = no evidence of inflammation; 1 = minor inflammation (metatarsal phalanges joints, individual phalanx, or localized edema); 2 = moderate swelling but localized to either dorsal or ventral surface of paw; and 3 = highly inflamed swelling on all aspects of paw. Maximum score is 12. The procedure was the same for experiments targeting the right leg, except that the skin above the anterior and posterior regions of the thigh was shaved, and the sciatic and femoral nerves were each targeted with 2 minutes of US stimulation. Supplementary Fig. 3a shows an example of the 1 MHz US transducer coupled to the mouse by the focusing cone that is filled with degassed water. The cone tip is lowered into place so that the tip of the cone is just barely in contact with the skin during US or Sham-US. To target the spleen, the shoulder and hip joints were used as landmarks. Repeated dissections of euthanized mice confirmed that the spleen is consistently located halfway between the shoulder and hip joints of the animal, so these landmarks were subsequently used to target the spleen. Therefore, the tip of the US focusing cone was positioned at the midpoint between the shoulder and hip joint of the animal’s left flank (Supplementary Figure 3b), and 5mm anterior to the spine (Supplementary Figure 3a). The cone was angled slightly caudally, to allow the US pressure wave to follow the angle of the spleen (Supplementary Figure 3b). Some stimulations, referred to as “contra-spleen” were positioned identically to the spleen-targeted setup, except that the cone was placed on the animal’s right flank instead of left flank, contralateral to the spleen. These contralateral stimulation cases administered the same US energy to the animals except that the spleen was not targeted. Bodyweight was measured daily before anesthetizing the animal by placing the animal in a plastic weighing container on a digital scale (accuracy 0.1g). In order to minimize any circadian effects in which time of day could influence the effectiveness of US treatment, the order of the animals in terms of anesthetization and stimulation was changed every day.

*Individually Plotted Preventative Arthritis Experiments.* Supplementary Fig. 4 shows the pooled data from the preventative arthritis experiments (Fig. 2a,b) plotted as individual experiments. Change in ankle thickness (a, c, e) and clinical score (b, d, f) is shown for days 0 to 7 of each experiment. All experiments used the same US parameters applied from day -1 through 6; 1 MHz US at 350 kPa, 1s on/5s off bursts with the shallow US-focusing cone to target the spleen in animals with arthritogenic serum transfer at day 0.

*Arthritogenic Serum Transfer Validation.* On the final day of the experiment after CO_2_ euthanasia, terminal blood was collected from each animal and spun in a centrifuge. A sample of that serum was separated for an anti-Glucose-6-Phosphate Isomerase (anti-GPI) ELISA analysis as previously described (S1). Anti-GPI autoantibodies, transferred to the animal on day 0, are responsible for causing arthritis in the mice. If an animal’s terminal blood did not contain an elevated anti-GPI level above the level measured from wildtype C57BL/6 mice, it was considered as having received an unsuccessful injection and was excluded from the study. Out of the 276 mice used in the experiments for this report, five were found to have low anti-GPI levels and were excluded from the analysis.

*US Duration Linear Regression*. The ankle thickness and clinical scores follow a duration-dependent trend that is statistically significant (p < 0.0001 and p = 0.0460, respectively in Supplementary Fig. 5). These results demonstrate that the therapeutic benefits of US stimulation are dose-dependent and that longer US treatment per day results in a higher benefit.

*Disease-Driven Weight Loss.* The bodyweight of each animal was measured just before the daily stimulation session using a digital scale (Ohaus Model CL 201, 0.1g resolution). Supplementary Fig. 6 shows that disease driven weight loss follows an inverse relationship with US stimulation duration. This trend suggests that animals that received more US treatment and were clinically improved were more inclined to eat and drink compared to the animals that received shorter durations of US stimulation. The linear regression of the normalized change in bodyweight at day 7 resulted in a p value of 0.0077.

*Individually Plotted Therapeutic Arthritis Experiments.* Supplementary Fig. 7 shows the pooled data from the therapeutic arthritis experiments (Fig. 3e,f) plotted as individual experiments. Change in ankle thickness (a, c) and clinical score (b, d) is shown for days 3 to 7 of each experiment. All experiments used the same US parameters applied from day 3 through 6, which was 1 MHz US at 350 kPa, 1s on/5s off bursts with the shallow US-focusing cone to target the spleen in animals with arthritogenic serum transfer at day 0.

*Cohort Sorting Protocol.* For most experiments, animals were randomly placed into each cohort without bias on day -1. For the experiments in which US treatment was initiated 3 days post serum injection (Fig. 3e,f, and Supplementary Fig. 7), animals were sorted on day 3 based on their ankle thickness. Cohorts were formed based on a running average of ankle swelling. The sorting was accomplished by randomly selecting and anesthetizing each animal, measuring their ankle thickness, and comparing to each cohort’s mean thickness. A mouse was assigned to either the US or sham-US group by whichever group’s mean ankle thickness was most different from that chosen animal’s. This had the desired effect of equalizing the mean ankle thickness from both cohorts, resulting in a final mean ankle thickness that was almost identical between groups.

*scRNA-seq Experimental Methods, Sample Preparation and Cell Sorting.* Sixteen mice were used for the scRNA-seq experiment, with an n = 4 in each of the four experimental cohorts. Animals either received arthritogenic serum or sham serum (from non-arthritogenic donors) and underwent either 1 MHz US at 350 kPa using 1s on/5s off bursts or sham-US for 12 minutes per day from days -1 to 6. A shallow US-focusing cone (see Supplementary Fig. 2) was used to target the spleen. The resulting four cohorts were: (1) Sham serum + sham-US, (2) Sham serum + 1 MHz US, (3) Arthritogenic serum + sham-US, and (4) Arthritogenic serum + 1 MHz US. Spleens were dissected from each mouse approximately 2.5 – 4 hours post-treatment on the seventh day of treatment and samples were kept on ice for the entirety of the experiment. Spleens were processed into single cell suspensions, and homogenization and staining were performed in 2% BSA in PBS supplemented with 5 mM EDTA. Antibodies used for cell sorting included APC CD45.1 (BioLegend Cat. No. 109813, stained at 1ng/uL per 100 million cells), and SYTOX Green (LifeTechnologies Cat. No S34860, stained at 30nM per 100 million cells). Sorting gating strategy selected for live, singlet CD45 positive leukocytes and cells were sorted on BD FACSAria II (BD Biosciences) into 50% FBS in PBS.

*scRNA Library Preparation, Sequencing and Statistical Analyses.* Cell count and viability for samples were determined immediately after cell sorting. Cell viability for each sample ranged between 60-85% and cells libraries were made from a target of 10,000 cells per sample. Libraries were made via the 10X Genomics Chromium Single Cell 3’ Library & Gel Bead Kit (v2) per manufactures instructions. Samples were run on two lanes of Illumina NovaSeq Sequencer resulting in (per sample) median number of: cells = 6166, reads = 72,901, and genes detected = 5,148. Next reads were mapped to the mm10 mouse genome using ‘Cell Ranger’. Analyses were then performed using R version 3.5.1 (2018-07-02) and primarily using packages ‘Seurat’, ‘Matrix’ and visualization using ‘ggplot2’ (S2). To process data, we first only considered expressed genes that were detected in at least 0.1% of the cells. Based on comparisons of the profiles of the 16 samples, cells were filtered, keeping only those cells with number of genes detected per cell > 200 and < 3000, and percent mitochondrial genes < 0.075. Samples were then log normalized, and scaled whereby number and two variables (unique molecular identifiers (UMIs) and percent mitochondrial genes) were regressed out. Clusters were determined using the first 8 principal components and graphed using tSNE dimensional reduction for each sample. Based on clustering and marker genes defining each cluster, bulk T cells, B cells, Myeloid cells and NK cells were identified (using primarily expression of *Cd3g*, *Ms4a1*, *Fcer1g* and *Ncr1*, respectively) and assigned. Differential expression analysis was then performed using the Wilcox rank sum test on T cells and B cells between sham and US treatments within either arthritic or non-arthritic mice. T cells and B cells were specifically evaluated in this study due to their relevance to the proposed mechanism of action for electrical stimulation or US stimulation of the anti-inflammatory pathway through the spleen based on previous studies (S3, S4). Future analyses will be performed on the other cell types to further elucidate the mechanisms and pathways involved with the anti-inflammatory effects elicited by US targeting of the spleen. Expression of these significantly differentially expressed gene are reported in T cells and B cells with an adjusted p-value using the Bonferroni correction.

*Ultrasound Heating Effects.* To estimate the amount of heating resultant from US exposure in mice, a computational model of the US transducer was first constructed in COMSOL Multiphysics v5.2 (COMSOL, Burlington, MA) to match the measured pressure profile of the transducer with the focusing cone, and then applied to a simplified model of the mouse to calculate the resultant pressure, intensity and heat generation due to US. These methods are similar as done previously by Mueller et al [S5]. In brief, a two-dimensional computer geometry with axial symmetry was created based on the geometry of the US focusing cone and then adjusted to match the pressure profile and amplitude observed in acoustic tank measurements, recreating the physical transducer in a computational environment. Following agreement between the computational model and free water measurements in the acoustic test tank, a simplified model of the mouse was then directly coupled to the aperture of the focusing cone. The mouse was represented as a homogenous cylinder of muscle that was 1 inch in diameter and height, with 0.25 inch rounded corners. The material properties of the mouse were defined as the average values of muscle [S6], with acoustic property values taken at a frequency of 1 MHz. Additionally, cooling due to blood perfusion in the mouse was neglected, and thus heating in the model represents a conservative overestimate erring on the side of overheating.

Supplementary Fig. 9a shows the resultant heating profile in a cross section of the mouse model after 1 second of continuous US exposure, mimicking the optimal parameters for US detailed in this work. Note that the peak increase of temperature of about 0.12°C is localized near the site of the aperture of the cone at the top-center of the cross section. While there is a lower second peak at the opposite end of the mouse, this local maximum is due to the simplified flat back surface of the mouse consistently reflecting US back onto the main axis of US propagation and would be reduced in an actual mouse where perfectly flat surfaces are not present. Supplementary Fig. 9b shows the heating over time at a depth of 1 mm and 3 mm along the midline from the aperture of the cone. Note that while there is heating during the initial 1 second period of US exposure, much of the localized heat is dissipated to surrounding tissues during the remaining 4 seconds of stimulus exposure when no US is being delivered. Overall, our model conservatively estimates that we would expect minimal heating over the 5 second period of US stimulation (less than 0.03°C in the region of peak effects after 5 seconds), which will likely dissipate due to blood perfusion in the mouse to result in minimal or no net heating.

**Supplementary Figures**

**Supplementary Figure 1 | Time course of arthritis in untreated animals. a,b**, Two separate arthritic cohorts were injected on day 0 and again on day 2 with 150 μl of K/BxN serum and each cohort received different batches of arthritogenic serum to test reliability. The third cohort did not receive arthritogenic serum. n = 10 for Arthritogenic Cohort 1, n = 10 for Arthritogenic Cohort 2, and n = 3 for the Non-Serum Cohort. **c,d**, Animals were injected with 300 ul of arthritogenic serum at day 0. n = 6. Means and SEM are shown for all curves.


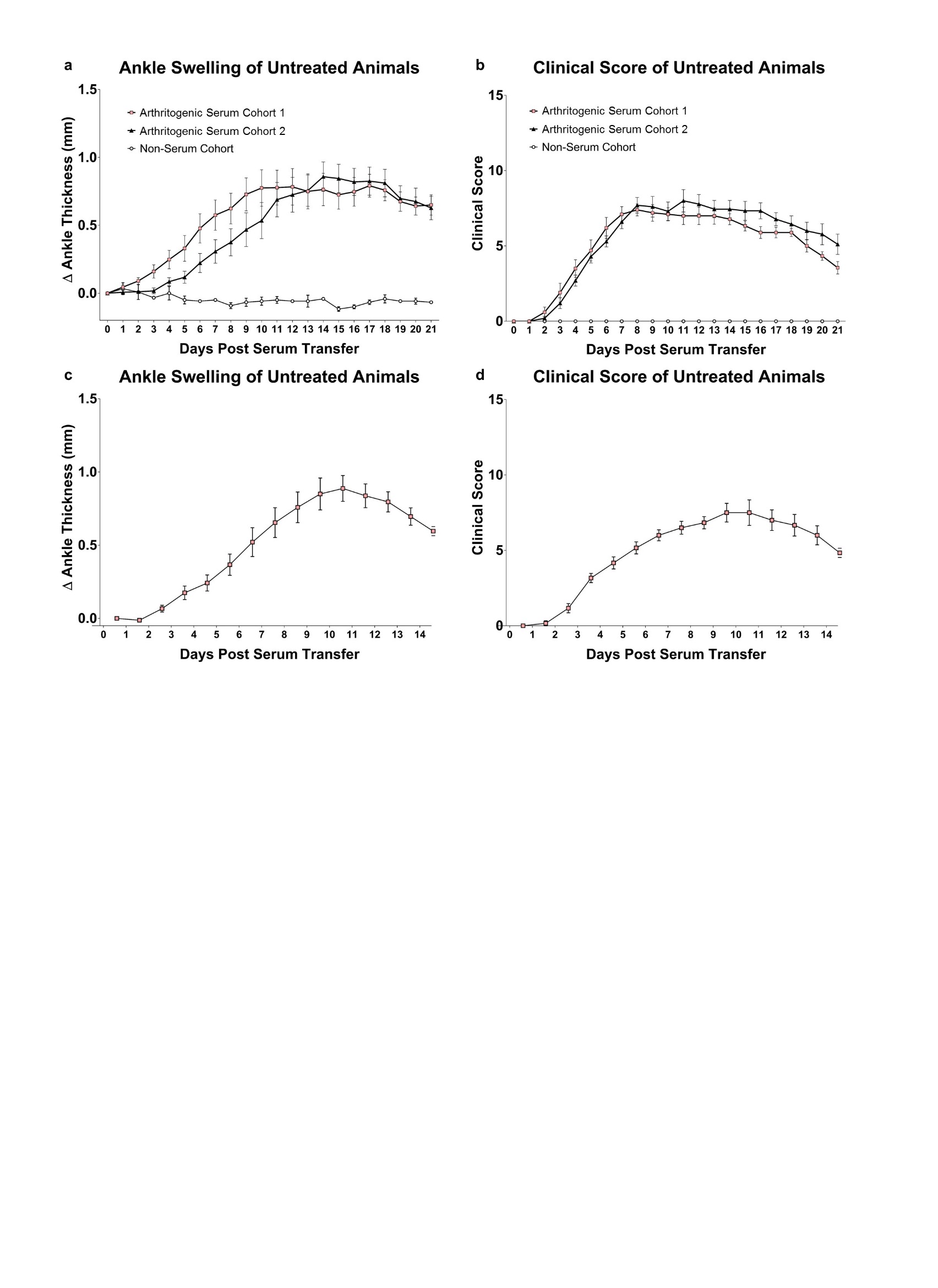

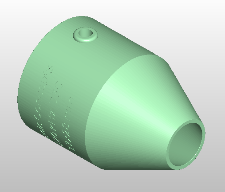

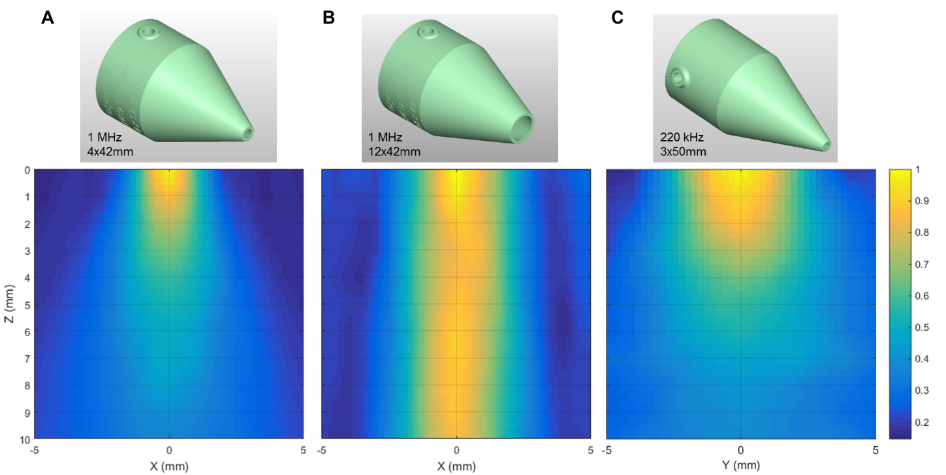


**Supplementary Figure 2 | Ultrasound (US) pressure field maps of the transducers used for noninvasive US stimulation.** Custom coupling cones were designed and printed for the 1 MHz transducer **(a, b)** and the 220 kHz transducer **(c, d)** that either provided shallow sonication regions **(a, c)** or deeper sonication regions **(b, d).** US pressures were measured by a hydrophone in degassed water. The origin in the Z axis corresponds to measurements made at an offset of 1 mm from the cone tip. Maximum pressure is normalized to a value of 1. Higher pressure regions correspond to yellow on the normalized scales.


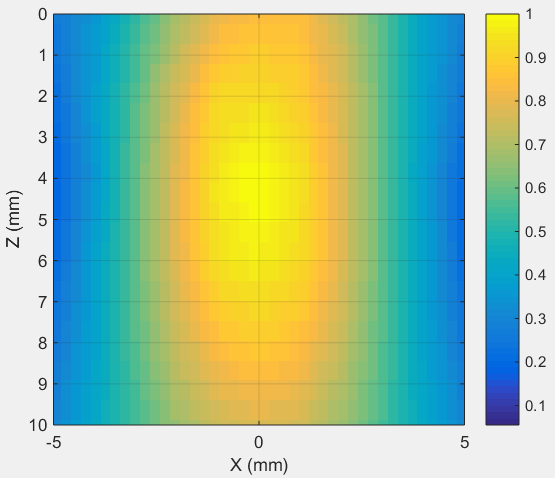


220 kHz

17x28mm

**X (mm)**

**X (mm)**

**X (mm)**

**X (mm)**

**Z (mm)**

**a. Shallow 1 MHz cone b. Deep 1 MHz cone c. Shallow 220 kHz cone d. Deep 220 kHz cone**


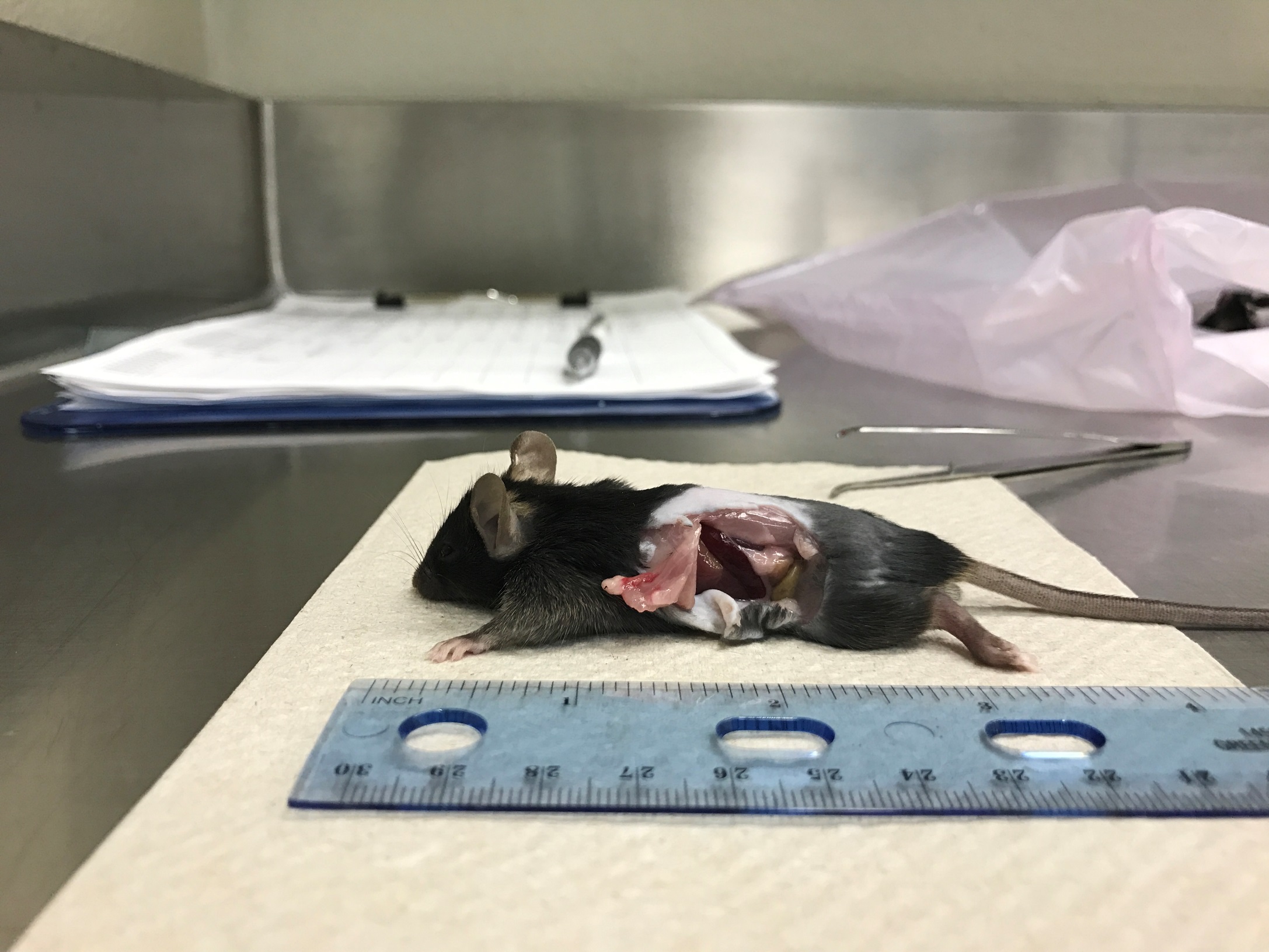


**Supplementary Figure 3 | Focusing US stimulation on the spleen. a**, The US transducer and focusing cone are coupled to the mouse with ultrasound gel. The US cone is positioned to target the murine spleen based on the beam profile characterizations described in Supplementary Figure 2. **b**, Repeated dissections of euthanized mice confirmed that the spleen is consistently located halfway between the shoulder and hip joints of the animal, so these landmarks were subsequently used to target the spleen.


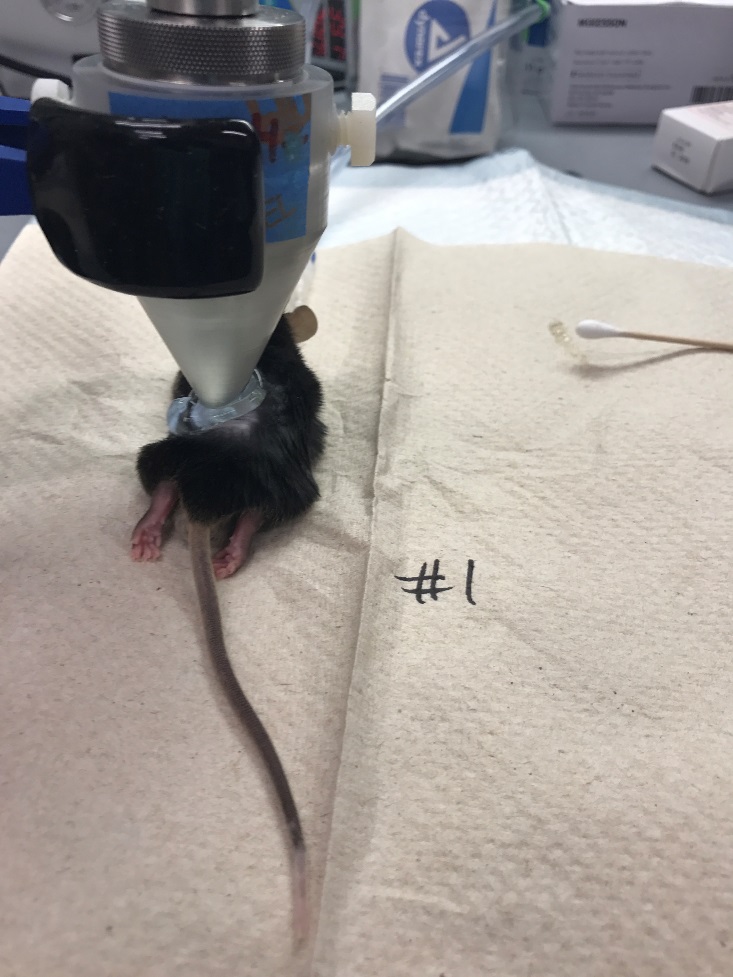


**a.**

**b.**

**Spleen**

**Shoulder Joint**

**Hip Joint**

**Midpoint**

**Supplementary Figure 4 | Individually Plotted Preventative Arthritis Experiments.**  Pooled data from the three preventative arthritis experiments (Fig. 3a,b) is shown plotted as individual experiments. In **a** and **b** (n = 8), **c** and **d** (n = 12), and **e** and **f** (n = 12), points indicate individual animals in the experiment. Change in ankle thickness **(a, c, e)** and clinical score **(b, d, f)** are shown for days 0 to 7 of each experiment. All experiments used the same US parameters applied from day -1 through 6, which was 1 MHz US at 350 kPa, 1s on/5s off bursts with the shallow US-focusing cone to target the spleen in animals with arthritogenic serum transfer at day 0.


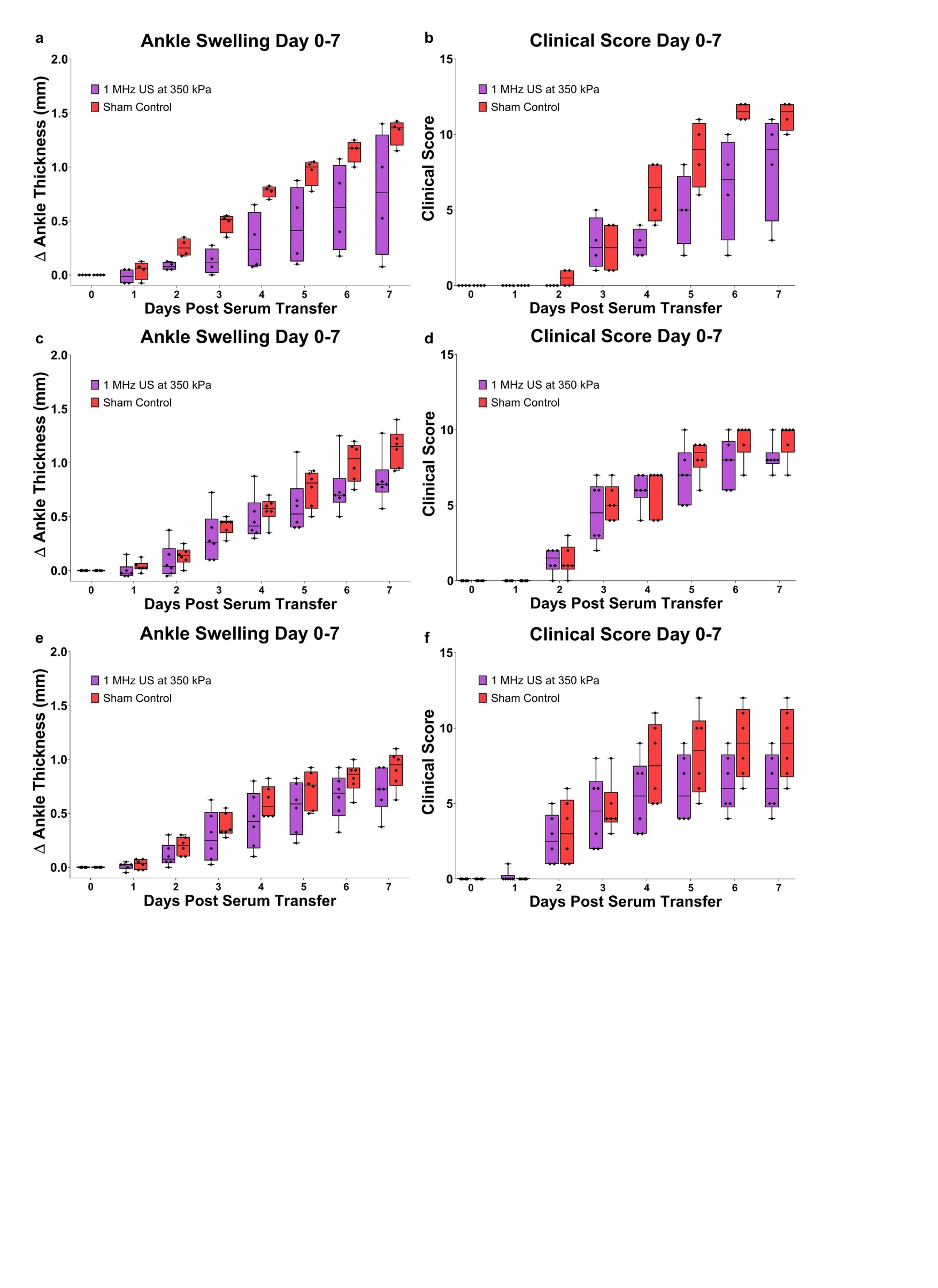


**Supplementary Figure 5 | Linear regression of ultrasound duration response.** Normalized 7th day measurements of ankle thickness **(a)** and clinical score **(b)** for the different durations of ultrasound treatment shown in Fig. 3a-d. 6, 12 and 20 min values were normalized to the pooled sham control data from Fig. 3a,b. The linear regression has a p <0.0001, R^2^ = 0.3839 and p = 0.0460, R^2^ = 0.1187 for ankle thickness and clinical score, respectively. Total n = 34.


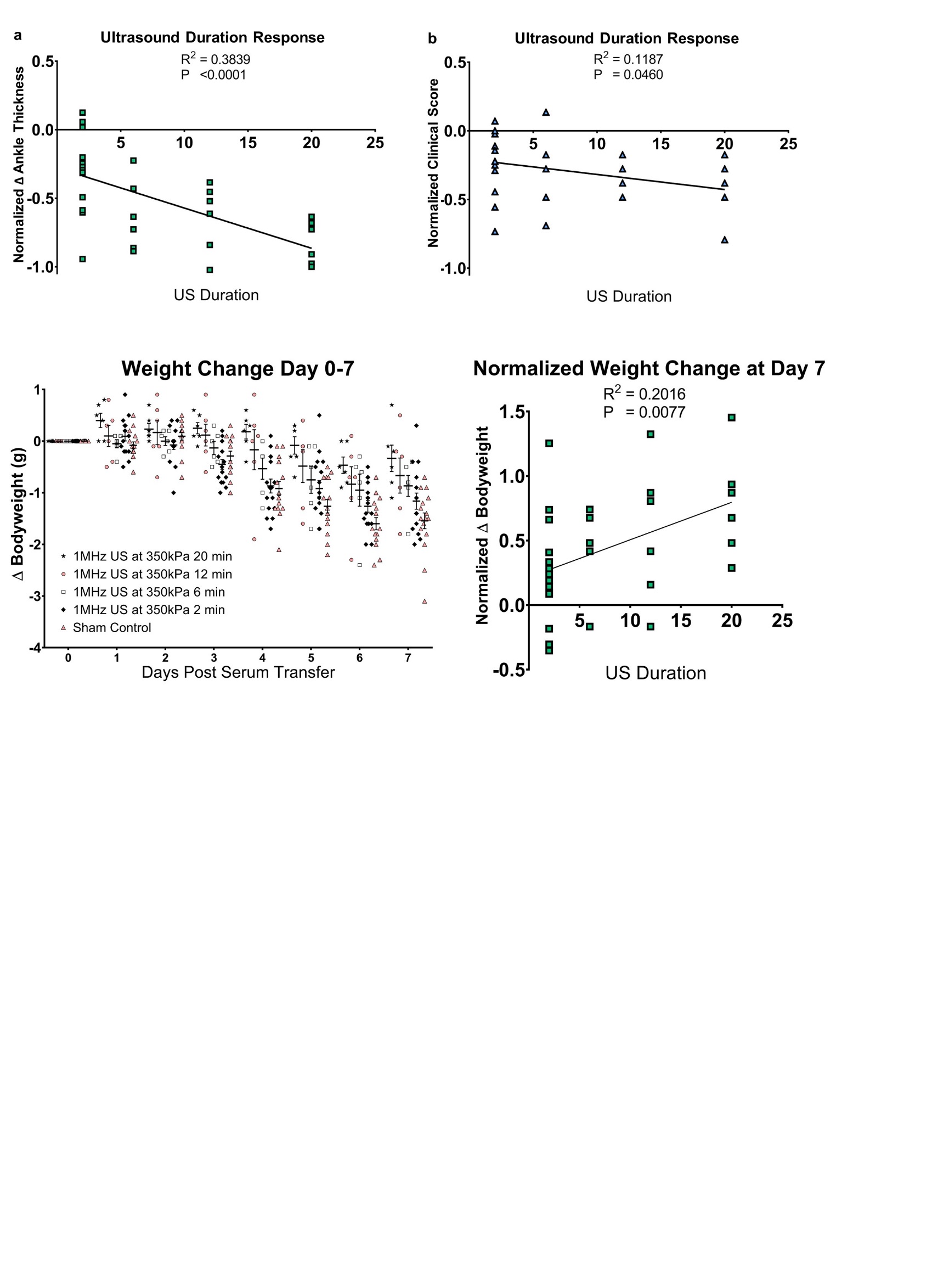


**Supplementary Figure 6 | Disease-driven weight loss.** Bodyweight correlated with the duration of US therapy delivered. **a**. The measured daily values for the change in bodyweight from baseline at day 0 are shown. These measurements are from the experiments shown in Fig. 3c,d. The US focusing cone was placed over the spleen of the sham control animals for 2 minutes but the US was not turned on. Total n = 50. **b**. Normalized 7^th^ day change in bodyweight is shown for 2, 6, 12 and 20 minute US stimulation durations. 2, 6, 12 and 20 min values were normalized to the pooled sham control data in **a**. The linear regression of this duration dose-response has a p = 0.0077 and R^2^ = 0.2016. Total n = 34.

**a. Change in Bodyweight Day 0-7 b. Normalized Change in Bodyweight at Day 7**


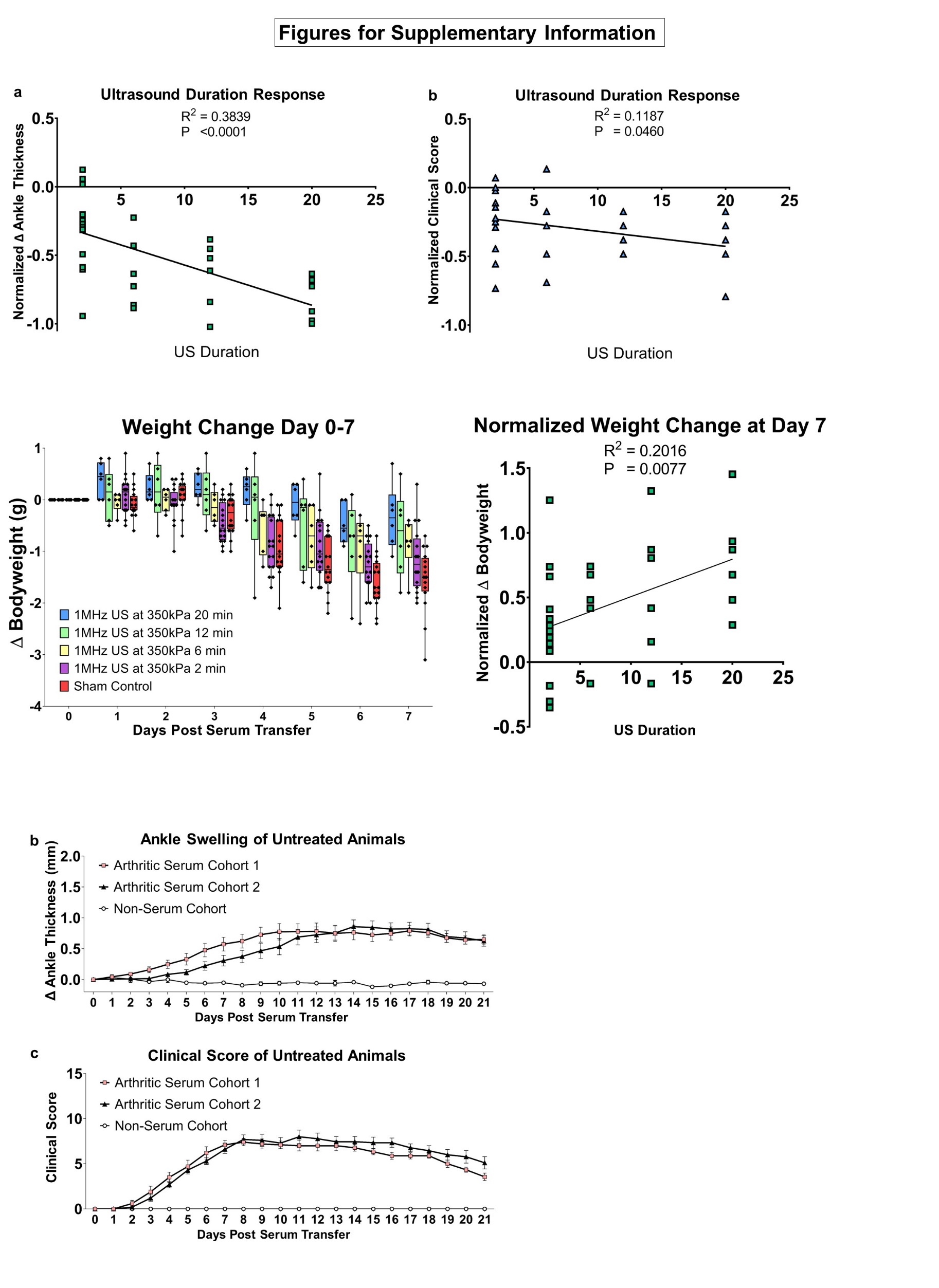

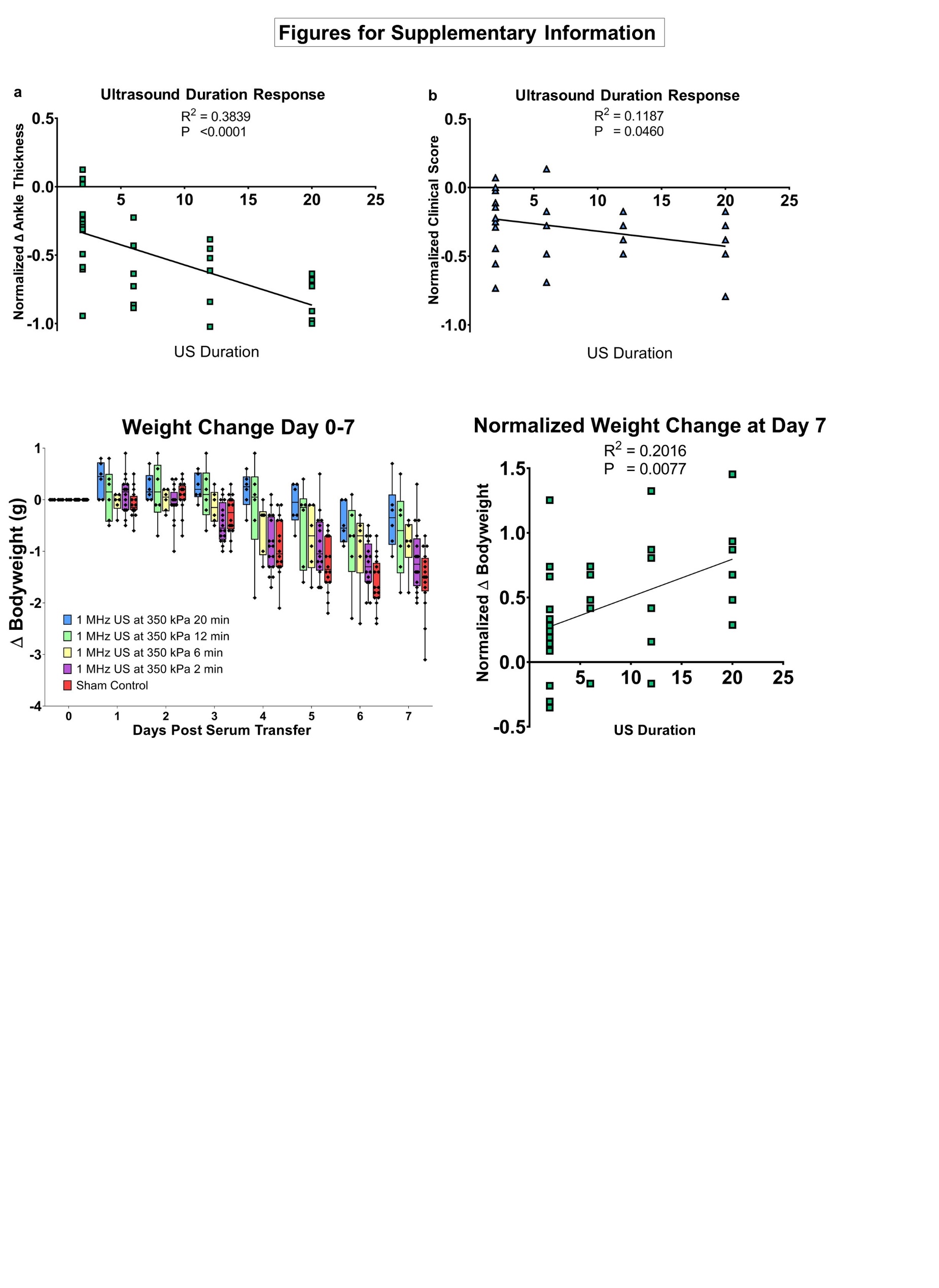

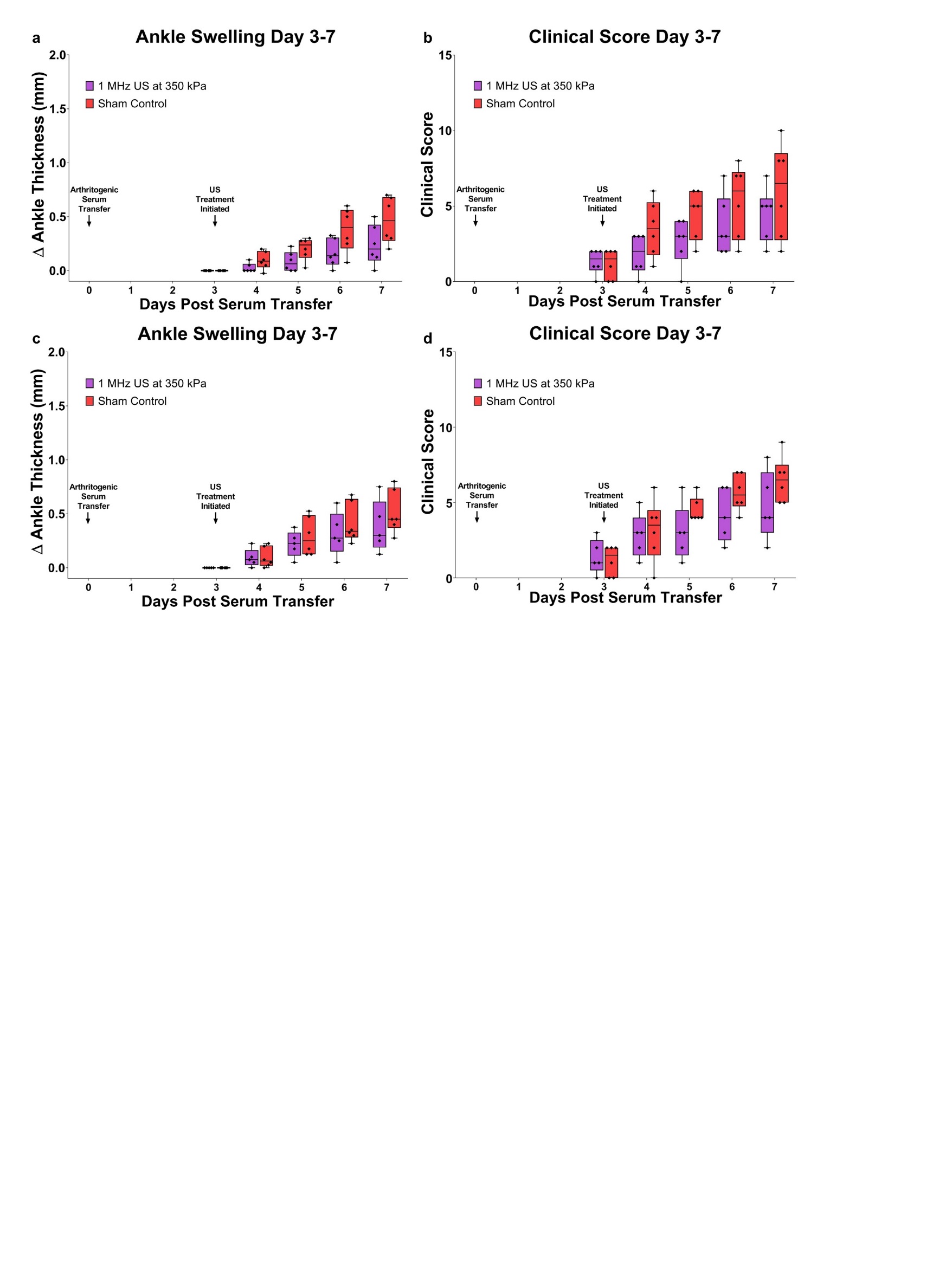


**Supplementary Figure 7 | Individually Plotted Therapeutic Arthritis Experiments.**  Pooled data from the two therapeutic arthritis experiments (Fig. 3e,f) is shown plotted as individual experiments. In **a** and **b** (n = 12), and **c** and **d** (n = 11), points indicate individual animals in the experiment. Change in ankle thickness **(a, c)** and clinical score **(b, d)** is shown for days 3 to 7 of each experiment. All experiments used the same US parameters applied from day 3 through 6, which was 1 MHz US at 350 kPa, 1s on/5s off bursts with the shallow US-focusing cone to target the spleen in animals with arthritogenic serum transfer at day 0.


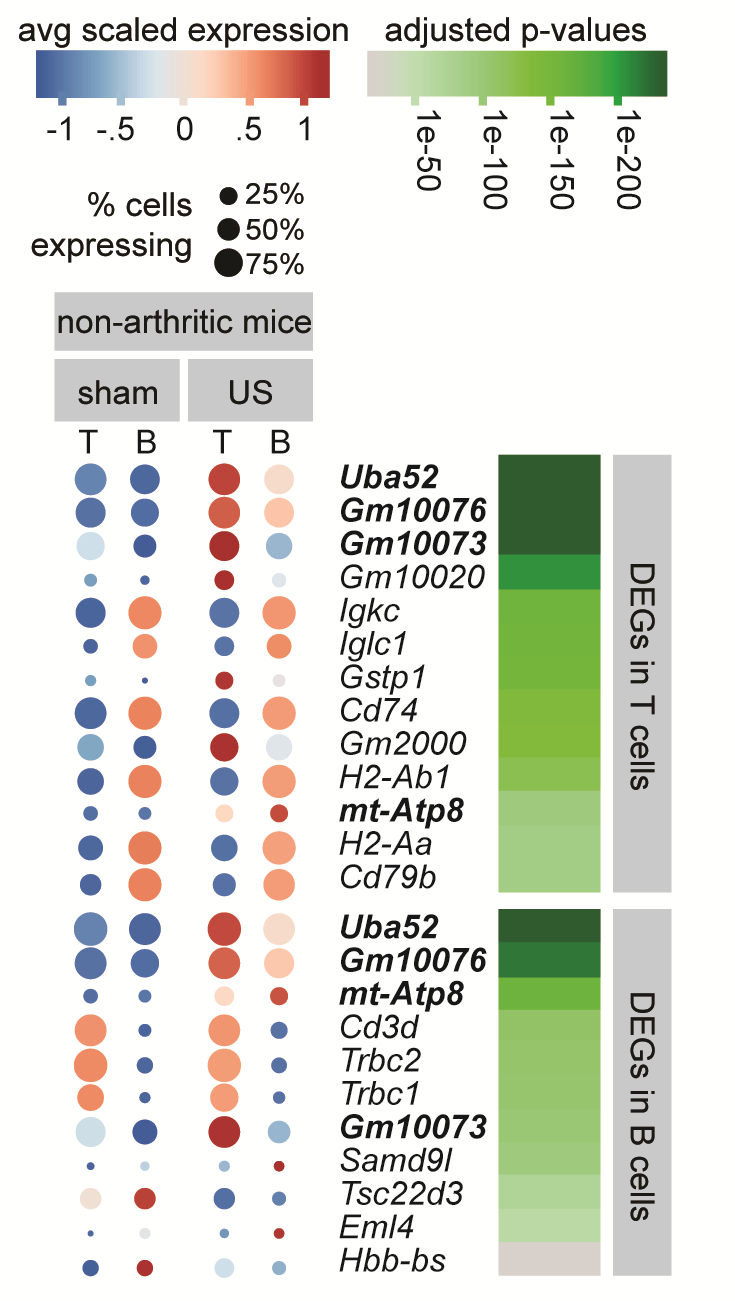


**Supplementary Fig. 8 | Differentially expressed genes in non-arthritic mice and ankle swelling of all animals from the RNA-sequencing experiment.** **a,** Dot plot showing differentially expressed genes (DEGs) are shown for sham-US and US-treated in non-arthritic mice in T cells (top list) and B cells (bottom list). The size of each circle represents the percent of cells within each cell type (T cells = T, B cells = B) which express the gene listed, and the color of each circle represents average scaled expression. Grey bars denote mouse treatment groups; Genes that are in bold are statistically significantly DEG in both T cells and B cells. P-values were calculated using a Wilcox rank sum test with Bonferroni correction.  **b,** US-treated arthritic animals had less day 7 ankle swelling than sham-US animals, while those animals receiving non-arthritogenic serum did not exhibit ankle swelling. Sham-US or US-treated (1 MHz US at 350 kPa, 1s on/5s off bursts) with the shallow US-focusing cone was used to target the spleen for 12 min daily on days -1 through 6. Four mice were in each cohort, with a total n equal to 16.

**a. Differentially Expressed Genes b. Day 7 Ankle Swelling of RNA-seq Mice**


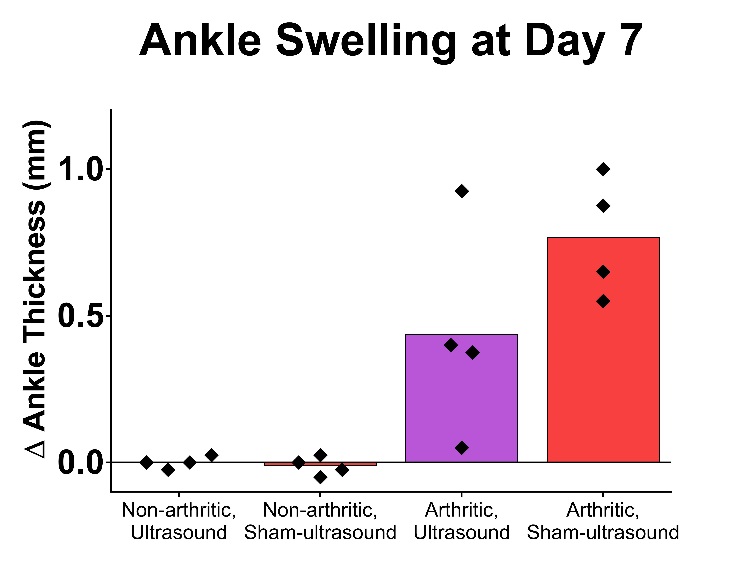

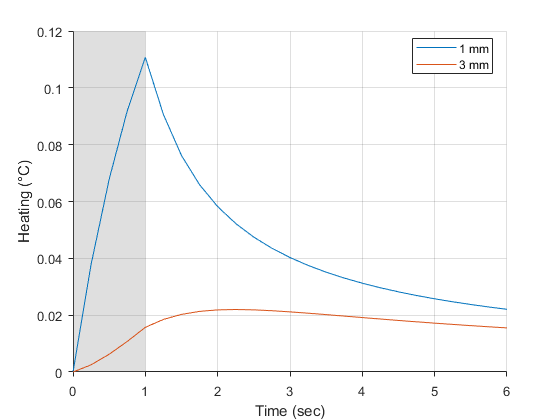

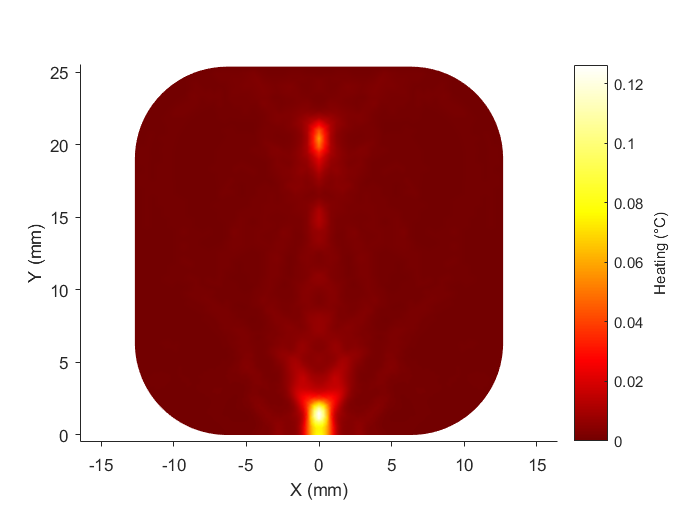


**Supplementary Figure 9 |** **Heating due to US in a simplified mouse model.** **a**, Cross section of heating in the mouse model after 1 second of US exposure. Note that peak heating is localized near the aperture of the cone at the top center of the cross section. **b**, Time course of heating at points 1 mm and 3 mm from the cone aperture midline. 1 second of US stimulation is indicated in gray. Note that 5 seconds after the ultrasound period ends (before the next US exposure), much of the generated heat in the peak region (blue line) has dissipated to surrounding tissue.

**a. Cross Section of Heating b. Time Course of Heating**

Heating (°C)

Heating (°C)

Y (mm)

X (mm)

Time (seconds)


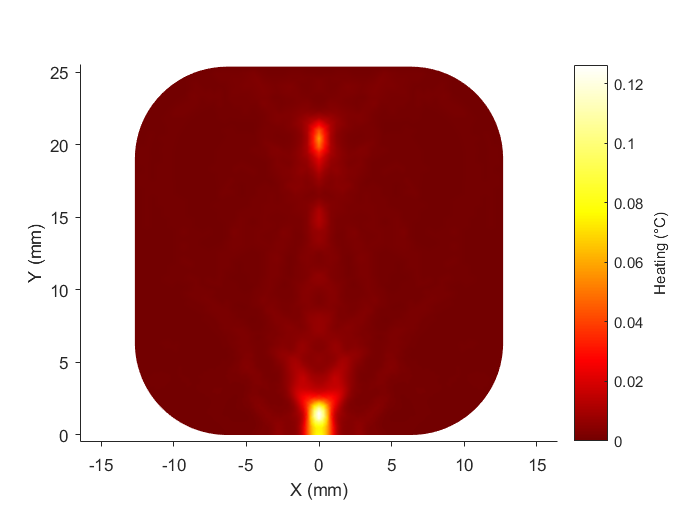


0

5

10

15

20

25

**Supplementary Tables**

**Supplementary Table 1 | Mouse spleen depth and dimensions (measured in mm)**

|  | Spleen Depth | Spleen Length | Spleen Width | Spleen Thickness |
| --- | --- | --- | --- | --- |
| Mean | 0.95 | 13.00 | 3.56 | 1.94 |
| STD | 0.03 | 1.07 | 0.42 | 0.18 |
| SEM | 0.01 | 0.38 | 0.15 | 0.06 |
| Number of mice | 8 | 8 | 8 | 8 |

**Supplementary Table 2 | Differentially expressed genes in T and B cells between US and sham-US treatments in both arthritic and healthy mice**

| T Cell Response | | | | | |
| --- | --- | --- | --- | --- | --- |
| Arthritogenic Injection, US Versus Sham-US DEGs | | | | | |
| Gene Name | p value | average logFC | ratio of cells US | ratio of cells sham | adjusted p value |
| *Gm26917* | 2.98e-234 | 0.490306873 | 0.611 | 0.471 | 3.95e-230 |
| *mt-Atp8* | 2.00e-200 | 0.456828801 | 0.291 | 0.134 | 2.65e-196 |
| *Gm42418* | 5.60e-106 | 0.311662528 | 0.331 | 0.21 | 7.41e-102 |
| *Junb* | 5.18e-103 | 0.424524265 | 0.453 | 0.34 | 6.86e-99 |
| *AY036118* | 1.33e-92 | 0.254744523 | 0.2 | 0.104 | 1.77e-88 |
| *Jun* | 4.57e-86 | 0.349508016 | 0.267 | 0.167 | 6.05e-82 |
| *Ssh2* | 3.81e-49 | 0.257171549 | 0.201 | 0.133 | 5.04e-45 |
| *Iglc1* | 1.80e-06 | 0.315991003 | 0.13 | 0.15 | 2.39e-02 |
| Sham Injection, US Versus Sham-US DEGs | | | | | |
| Gene Name | p value | average logFC | ratio of cells US | ratio of cells sham | adjusted p value |
| *Uba52* | 0 | 0.638863128 | 0.915 | 0.915 | 0.0000000 |
| *Gm10076* | 0 | 0.57327321 | 0.86 | 0.795 | 0.0000000 |
| *Gm10073* | 0 | 0.449484199 | 0.793 | 0.735 | 0.0000000 |
| *Gm10020* | 1.61e-255 | 0.408371414 | 0.338 | 0.154 | 2.12e-251 |
| *Igkc* | 1.09e-194 | 0.356840293 | 0.761 | 0.839 | 1.44e-190 |
| *Iglc1* | 1.84e-193 | 0.300454414 | 0.325 | 0.162 | 2.43e-189 |
| *Gstp1* | 9.72e-185 | 0.309051198 | 0.244 | 0.105 | 1.28e-180 |
| *Cd74* | 1.25e-167 | 0.300197471 | 0.806 | 0.859 | 1.65e-163 |
| *Gm2000* | 1.49e-160 | 0.255259259 | 0.728 | 0.614 | 1.96e-156 |
| *H2-Ab1* | 1.43e-144 | 0.302663372 | 0.675 | 0.635 | 1.89e-140 |
| *mt-Atp8* | 2.29e-108 | 0.369786946 | 0.276 | 0.167 | 3.02e-104 |
| *H2-Aa* | 1.42e-99 | 0.25858929 | 0.603 | 0.524 | 1.87e-95 |
| *Cd79b* | 2.17e-98 | 0.253971307 | 0.486 | 0.36 | 2.86e-94 |

| B Cell Response | | | | | |
| --- | --- | --- | --- | --- | --- |
| Arthritogenic Injection, US Versus Sham-US DEGs | | | | | |
| Gene Name | p value | average logFC | ratio of cells US | ratio of cells sham | adjusted p value |
| *Gm26917* | 7.4e-183 | 0.476842561 | 0.523 | 0.391 | 9.76e-179 |
| *mt-Atp8* | 8.4e-161 | 0.464388437 | 0.259 | 0.127 | 1.12e-156 |
| *Ddx5* | 1.1e-130 | 0.251816042 | 0.848 | 0.767 | 1.47e-126 |
| *AY036118* | 4.71e-75 | 0.259483484 | 0.165 | 0.087 | 6.23e-71 |
| *Gm42418* | 4.14e-72 | 0.34145544 | 0.266 | 0.176 | 5.49e-68 |
| *Mndal* | 2.68e-52 | 0.262613986 | 0.372 | 0.291 | 3.55e-48 |
| *Jun* | 8.7e-47 | 0.315790254 | 0.198 | 0.134 | 1.15e-42 |
| *Eml4* | 2.3e-42 | 0.269609477 | 0.154 | 0.099 | 3.04e-38 |
| *Macf1* | 2.27e-41 | 0.268537566 | 0.2 | 0.139 | 3e-37 |
| *Iqgap1* | 1.02e-40 | 0.252054327 | 0.317 | 0.25 | 1.35e-36 |
| *Actb* | 7.72e-40 | -0.321662752 | 0.973 | 0.977 | 1.02e-35 |
| *Ssh2* | 1.32e-38 | 0.25661227 | 0.123 | 0.075 | 1.75e-34 |
| Sham Injection, US Versus Sham-US DEGs | | | | | |
| Gene Name | p value | average logFC | ratio of cells US | ratio of cells sham | adjusted p value |
| *Uba52* | 2.49e-292 | 0.480297963 | 0.827 | 0.828 | 3.28e-288 |
| *Gm10076* | 2.72e-272 | 0.447661149 | 0.769 | 0.692 | 3.58e-268 |
| *mt-Atp8* | 3.19e-198 | 0.528080302 | 0.271 | 0.133 | 4.21e-194 |
| *Cd3d* | 1.54e-130 | 0.293536689 | 0.243 | 0.127 | 2.02e-126 |
| *Trbc2* | 2.65e-125 | 0.338488486 | 0.317 | 0.192 | 3.49e-121 |
| *Trbc1* | 2.13e-123 | 0.251464096 | 0.191 | 0.09 | 2.80e-119 |
| *Gm10073* | 5.84e-116 | 0.310186003 | 0.571 | 0.471 | 7.69e-112 |
| *Samd9l* | 1.63e-106 | 0.332854456 | 0.173 | 0.086 | 2.14e-102 |
| *Tsc22d3* | 1.53e-74 | -0.318595313 | 0.256 | 0.342 | 2.01e-70 |
| *Eml4* | 1.00e-57 | 0.273775021 | 0.16 | 0.097 | 1.32e-53 |
| *Hbb-bs* | 6.73e-08 | -0.255690676 | 0.228 | 0.193 | 8.86e-04 |

Adjusted p-values in Supplementary Table 2 were calculated using a Wilcox rank sum test with Bonferroni correction.

**Supplementary References**

1. Binstadt, B.A. et al. The same systemic autoimmune disease provokes arthritis and endocarditis via distinct mechanisms. Proc Natl Acad Sci U S A 106, 16758-16763 (2009).

2. Satija, R., Farrell, J.A., Gennert, D., Schier, A.F. & Regev, A. Spatial reconstruction of single-cell gene expression data. Nat Biotechnol 33, 495-502 (2015)

3. Andersson, U. & Tracey, K.J. Neural reflexes in inflammation and immunity. J Exp Med 209, 1057-1068 (2012).

4. Mina-Osorio, P. et al. Neural signaling in the spleen controls B-cell responses to blood-borne antigen. Mol Med 18, 618-627 (2012).

5. Mueller, J.K., Ai, L., Bansal, P. & Legon, W. Computational exploration of wave propagation and heating from transcranial focused ultrasound for neuromodulation. J Neural Eng 13, 056002 (2016).

6. Hasgall PA, Di Gennaro F, Baumgartner C, Neufeld E, Lloyd B, Gosselin MC, Payne D, Klingenböck A, Kuster N, “IT’IS Database for thermal and electromagnetic parameters of biological tissues,” Version 4.0, May 15, 2018, DOI: 10.13099/VIP21000-04-0.
